# Quantum-aware secure blockchain intrusion detection system for industrial IoT networks

**DOI:** 10.1038/s41598-025-31985-0

**Published:** 2025-12-11

**Authors:** Nasir Hussain, Shuaiyong Li, Altaf Hussain, Zahid Ullah, Mona Jamjoom

**Affiliations:** 1 School of Computer Science and Technology, Chongqing University of Posts and Telecommunications, Chongqing, 400065 China; 2 Key Laboratory of Industrial Internet of Things and Networked Control, Ministry of Education, Chongqing University of Posts and Telecommunications, Chongqing, 400065 China; 3 Information Systems Department, College of Computer and Information Sciences, Imam Mohammad Ibn Saud Islamic University (IMSIU), Riyadh, 11432 Saudi Arabia; 4 Department of Computer Sciences, College of Computer and Information Sciences, Princess Nourah bint Abdulrahman University, Riyadh, 11671 Saudi Arabia

**Keywords:** Quantum optimization, Quantum key distribution, Industrial IoT, Intrusion detection system, IIoT cyber security, QASB-IDS, Engineering, Mathematics and computing

## Abstract

The Industrial Internet of Things (IIoT) integrates sensors, actuators, controllers, and gateways across critical industrial sectors such as manufacturing, energy, oil and gas, and transportation. The convergence of operational technology (OT) and Information Technology (IT) has enabled real-time automation, predictive maintenance, and large-scale process optimization. However, IIoT infrastructures are increasingly exposed to sophisticated cyberattacks, ranging from ransomware and Advanced Persistent Threats (APTs) to false data injection and denial-of-service (DoS) campaigns. Moreover, the emergence of quantum computing introduces an additional dimension of vulnerability, since widely deployed public-key cryptographic schemes such as RSA and elliptic-curve cryptography (ECC) will be rendered insecure under Shor’s algorithm, while Grover’s algorithm accelerates brute-force search against symmetric ciphers. In anticipation of this threat, the security of IIoT must evolve toward post-quantum resilience. This paper proposes a Quantum-Aware Secure Blockchain Intrusion Detection System (QASB-IDS), a novel hybrid framework that combines intrusion detection with post-quantum blockchain security and quantum key distribution (QKD). The system introduces a hierarchical consortium blockchain tailored to IIoT, ensuring tamper-proof and decentralized storage of intrusion detection alerts and anomaly model updates. The IDS employs a lightweight hybrid signature–anomaly detection approach that extracts features from IIoT traffic and classifies them using a CNN–LSTM model optimized for constrained devices using Edge-IIoTset, SWaT, and XIIoTID datasets. Model updates are securely aggregated with privacy guarantees and anchored on blockchain using lattice-based post-quantum signatures. Critical gateways additionally deploy QKD to protect session key distribution. Evaluation using recent industrial intrusion datasets demonstrates that QASB-IDS provides improved detection accuracy, low false alarms, and strong resilience against both classical and quantum-capable adversaries.

## Introduction

The Internet of Things (IoT) has revolutionized the way devices interact, enabling seamless connectivity and intelligence across physical objects. By embedding sensors, actuators, and computational capabilities into everyday devices, IoT allows real-time data collection, remote monitoring, and automated control. This interconnectivity has unlocked transformative opportunities in healthcare, smart cities, transportation, and industrial monitoring, facilitating predictive analytics, efficient resource management, and data-driven decision-making^1–3^. However, the rapid proliferation and heterogeneity of IoT deployments bring critical challenges in security, privacy, and interoperability, emphasizing the need for robust protective mechanisms^4–6^.

Building upon IoT, the Industrial Internet of Things (IIoT) extends connectivity and intelligence to industrial environments, integrating sensors, actuators, controllers, and gateways across sectors such as manufacturing, energy, oil and gas, and transportation. By converging operational technology (OT) with information technology (IT), IIoT enables real-time automation, predictive maintenance, and large-scale process optimization^7,8^. The adoption of IIoT has dramatically enhanced operational efficiency, reduced downtime, and improved situational awareness. Yet, these advantages come with increased vulnerability: IIoT infrastructures are inherently complex, interconnected, and resource-constrained, making them prime targets for sophisticated cyberattacks^9,10^.

Cybersecurity in IIoT faces unprecedented challenges. Industrial networks are increasingly threatened by ransomware, advanced persistent threats (APTs), false data injection, and distributed denial-of-service (DDoS) attacks. Traditional security mechanisms often fail to address the unique constraints of IIoT, including real-time operational requirements, limited computational resources on edge devices, and heterogeneous protocols and systems. Consequently, Intrusion Detection Systems (IDS) have become indispensable for safeguarding IIoT environments^2,11,12^. However, existing IDS solutions often struggle with scalability, high false-alarm rates, and adaptability to rapidly evolving threat landscapes^13–15^.

The emergence of quantum computing adds another critical dimension to IIoT security. Quantum algorithms, such as Shor’s algorithm, threaten widely deployed public-key schemes including RSA and elliptic-curve cryptography (ECC), while Grover’s algorithm accelerates brute-force attacks against symmetric encryption^16–18^. This imminent risk underscores the urgent need for post-quantum secure mechanisms in IIoT, ensuring that confidentiality, integrity, and availability are maintained even in the presence of quantum-capable adversaries^19–23^.

Blockchain technology offers promising solutions for IIoT security by providing decentralized, tamper-proof, and auditable storage of critical data^4,24,25^. Blockchain can secure IDS alerts, anomaly detection models, and audit logs against unauthorized modifications. However, conventional blockchain implementations are insufficient against quantum threats, necessitating the integration of quantum-aware or post-quantum blockchain mechanisms, such as lattice-based digital signatures and quantum key distribution (QKD), to future-proof IIoT infrastructures^17,18^.

In response to these challenges, we propose QASB-IDS: Quantum-Aware Secure Blockchain Intrusion Detection System, a hybrid framework that integrates lightweight IDS, post-quantum blockchain security, and QKD for IIoT environments. QASB-IDS leverages a CNN–LSTM model optimized for resource-constrained devices to classify IIoT traffic with high accuracy, while a hierarchical consortium blockchain ensures tamper-proof, decentralized storage of alerts and model updates. Model updates are securely aggregated with privacy guarantees and anchored on the blockchain using lattice-based post-quantum signatures. Critical gateways employ QKD for secure session key distribution, providing resilience against both classical and quantum-enabled threats. Evaluation on benchmark industrial datasets—including Edge-IIoTset, SWaT, and X-IIoTID—demonstrates superior detection performance, low false-alarm rates, and robust security against advanced adversaries.

The proposed QASB-IDS model is conceptualized as a multi-layered architecture. At the edge layer, IIoT devices collect and preprocess operational data, and a hybrid IDS extracts features from network traffic and industrial process data, performing anomaly and signature-based detection using a lightweight CNN–LSTM model. Detected alerts and incremental model updates are transmitted to the consortium blockchain layer, acting as a tamper-proof ledger for storing alerts, model parameters, and audit logs. All updates are signed using lattice-based post-quantum signatures, while critical gateways deploy QKD for secure session key exchange. At the aggregation layer, privacy-preserving techniques combine IDS model updates from multiple nodes before anchoring them on the blockchain, enabling collaborative learning without compromising sensitive industrial data. This integrated approach ensures real-time intrusion detection, secure model sharing, and post-quantum resilience, effectively bridging IIoT operational efficiency with next-generation cybersecurity.

The key contributions of this work are:

- We propose a lightweight signature–anomaly intrusion detection system that integrates a CNN–LSTM architecture optimized for IIoT devices. .
- We design a post-quantum blockchain framework using a hierarchical consortium structure to ensure tamper-proof storage of alerts and model updates. 
- We formulate quantum-resilient security mechanisms by incorporating lattice-based signatures and quantum key distribution to provide robust protection against quantum-enabled threats. 
- We propose a privacy-preserving model aggregation scheme that securely aggregates IDS model updates while anchoring them on the blockchain. 
- We demonstrate a comprehensive evaluation through experiments conducted on multiple industrial datasets, validating the system’s accuracy, efficiency, and resilience to quantum-era attacks. 

The remainder of the paper is organized as follows: Sect. 2 reviews related work in IIoT security, intrusion detection, blockchain, and post-quantum cryptography, Sect. 3 presents the proposed QASB-IDS architecture, detailing the IDS design, blockchain framework, and quantum security mechanisms, Sect. 4 describes the experimental setup, datasets, metrics, and evaluation results, and Sect. 5 concludes the paper and highlights future research directions.

## Literature review

### Surveys and foundations (IoT/IIoT IDS Landscape)

Comprehensive surveys catalog datasets, attack taxonomies, modeling choices, and evaluation pitfalls for IoT/IIoT IDS. Classic baselines and dataset issues (class imbalance, aging benchmarks, train/test leakage) are detailed in^2,3,10^. These reviews emphasize the tension between accuracy and deployability under constrained compute, memory, and energy budgets. They also highlight the growing need for privacy-preserving training (federation) and trustworthy model provenance. Recent broad reviews on FL-security for IDS^26^ and ML techniques for IoT IDS^1^ echo the call for robust, label-efficient, and explainable designs that can tolerate distribution shift.

### Centralized ML/DL IDS for IoT/IIoT

A large body of work leverages CNNs, LSTMs, and hybrids for flow-based traffic representation. CNN–LSTM and attention-augmented RNN variants improve sequence modeling of multivariate flows and temporal correlations, reporting strong accuracy on common datasets: see^6–8,15^(note^8^: duplicates the same title as itself)^14,27–30^,. These works show (i) the value of temporal structure, (ii) the gains from ensembles/feature engineering, and (iii) the importance of reducing false positives in operational settings. However, most remain centralized (single data silo), have limited treatment of privacy, and rarely consider adversarial manipulation of training or deployment.

### Federated learning (FL) for IDS

FL avoids raw data pooling by training local models on edge devices and aggregating updates centrally. Works in this line target transportation IoT^12^, healthcare/IoT^24^, and general IoT^5,11,13^. They document benefits for data governance and latency, but also the new attack surface—poisoning from malicious clients, gradient leakage, and aggregation bias—necessitating robust/secure aggregation and fairness controls. Reviews like^26^ underline that feature engineering and client reputation/validation are crucial to mitigate byzantine behaviors, while lightweight aggregation (e.g., feature-based or partial-model protocols) helps on constrained nodes^11^.

### Blockchain-assisted or Ledger-Anchored IDS

To provide tamper-evident provenance for alerts, models, and audit trails, several works combine IDS with blockchain. In healthcare IoT, FIDChain anchors federated IDS outcomes on chain to guarantee immutability and traceability^24^. Decentralized frameworks for CPS/IIoT also appear, targeting collaborative detection with privacy preservation^25^ and blockchain-backed FL for IoT^4^. These contributions improve non-repudiation and cross-domain trust but typically rely on classical cryptography and do not model quantum-era adversaries; they also seldom optimize for heterogeneous IIoT tiers (sensors → gateways → control centers).

### Explainability and streaming analytics

Operational adoption benefits from interpretable alarms and streaming readiness. XAI applied to streaming IoT data^27^ shows how feature attributions can help analysts triage alerts and tune thresholds. Ensemble methods for edge platforms^28^ tackle compute budgets and seek lower false-alarm rates through complementary learners, though they still require trustworthy update pipelines.

### Defensive ML for IDS

Protecting the IDS itself from poisoning/evasion is an active area. Models and algorithms for defending ML components within IDS are discussed in^31^, covering detection of anomalous model updates, adversarial training, and resilient aggregation. These approaches are needed in FL and multi-stakeholder deployments but are not yet standard in IoT/IIoT IDS pipelines.

### Quantum-aware security and QML for IDS

Quantum computing threatens widely deployed public-key cryptosystems; parallel work explores quantum-enhanced analytics for detection. A direct QML IDS is proposed in QML-IDS^21^, while quantum outlier-based IDS demonstrates QML signal separation on DDoS-like traffic^19^. Broader discussions on quantum-enhanced frameworks for IoT security appear in^17,18,20^; systematic perspectives on anomaly detection toward QML are given in^22^. A quantum-inspired GA + self-supervision pipeline for WSN/IoT is reported in^23^. Feasibility and design space for quantum cryptography (e.g., QKD, QBER) in IoT are reviewed in^16^. These works either (i) focus on analytics (QML) without end-to-end system security, or (ii) focus on crypto (QKD/QC) without IDS/FL integration. Crucially, post-quantum cryptography (PQC) adoption and hybrid quantum-classical trust anchors in consortium IIoT settings remain underexplored.

### End-to-end collaborative detection in CPS/IIoT

For industrial CPS, decentralized, privacy-preserving collaborative IDS frameworks are emerging^25^, but typically lack: (a) hierarchical designs tuned to IIoT tiers, (b) cryptographic agility against quantum adversaries, and (c) integrated provenance for both alerts and model updates. Reference^32^ proposes a blockchain-aided self-sovereign identity framework tailored for UAV and edge systems. Blockchain ensures decentralized identity verification, tamper-resistant credential management, and improved trust among IoT/IIoT entities—an essential feature for secure device-to-device communication. Reference^21^ extends this toward a comprehensive edge-based security architecture for UAV systems, addressing threats such as spoofing, replay attacks, and unauthorized data access. This aligns with broader IoT/IIoT security requirements for robust authentication and secure data flow. Reference^33^ integrates blockchain into a privacy-aware task distribution architecture for UAV communication systems. Blockchain provides immutable task records, decentralized coordination, and stronger accountability—mechanisms increasingly vital in industrial IoT task scheduling.

### Research gaps and motivation

Existing studies in IoT and IIoT intrusion detection primarily address system-level vulnerabilities, but few works approach the problem from an integrated, quantum-resilient perspective. Traditional blockchain-based IDS frameworks such as FIDChain^24^ and decentralized CPS frameworks^25^ provide immutability and cross-domain trust, yet they still depend on classical cryptographic primitives—RSA, ECC, or SHA—rendering them susceptible to quantum attacks once large-scale quantum computers emerge. Similarly, federated learning-based IDSs^5,11–13,26^ enable decentralized model training and protect data privacy, but they seldom incorporate blockchain provenance or quantum-secure cryptography. These approaches treat privacy, trust, and post-quantum resilience as separate design axes rather than a unified architecture. Deep learning-based intrusion detection models^6,14,27^^7,15^,–^8^ attain high accuracy on standard datasets through CNN, LSTM, and attention mechanisms; however, they rely on centralized data pipelines and computational resources unsuitable for heterogeneous IIoT hierarchies. Moreover, they ignore adversarial manipulation and byzantine client risks that federated settings amplify^11,26,31^. Even though blockchain integration enhances auditability, current schemes rarely log both intrusion alerts and model-update histories in a verifiable manner. As a result, traceability across the entire IDS lifecycle—data collection, training, deployment, and alert dissemination—remains incomplete. On the quantum front, recent research has explored quantum-enhanced intrusion detection and cryptography, such as quantum outlier analysis^19^, QML-IDS^21^, and QESIF^20^, as well as systematic analyses of QML for IoT security^16,22,23^. Yet these works emphasize either analytical performance (through quantum or hybrid machine learning) or quantum key distribution feasibility, without connecting these capabilities to end-to-end IIoT security management. They seldom address how post-quantum cryptography, quantum-safe key exchange, and blockchain immutability can operate together within industrial network constraints. Consequently, the literature lacks an architecture that is simultaneously *quantum-aware*,* privacy-preserving*,* and operationally lightweight* for IIoT.

The research gap therefore lies in the absence of a holistic framework that unites intrusion detection, federated learning privacy, blockchain-based provenance, and quantum-resilient cryptography within the industrial IoT domain. Existing methods fail to secure model aggregation and alert dissemination against quantum-capable adversaries, neglect hierarchical IIoT deployment realities, and overlook the need for verifiable audit trails of both alerts and model versions. The proposed QASB-IDS framework closes this gap by fusing post-quantum blockchain, quantum key distribution, and a hybrid CNN–LSTM intrusion detector into a unified, IIoT-specific architecture. It employs lattice-based digital signatures to anchor alerts and model updates on a consortium blockchain, ensuring immutability even under quantum attacks. Federated aggregation with differential privacy and robust aggregation counters malicious clients, while lightweight edge-optimized inference maintains accuracy and efficiency on constrained nodes. QKD secures session key exchange among critical gateways, delivering information-theoretic confidentiality. Through this integration, QASB-IDS transforms fragmented advances—deep learning-based detection, federated privacy, blockchain trust, and quantum security—into a cohesive, end-to-end defensive fabric capable of resisting both classical and quantum adversaries in industrial IoT networks.

## Proposed methodology

### System architecture

**Fig. 1:**
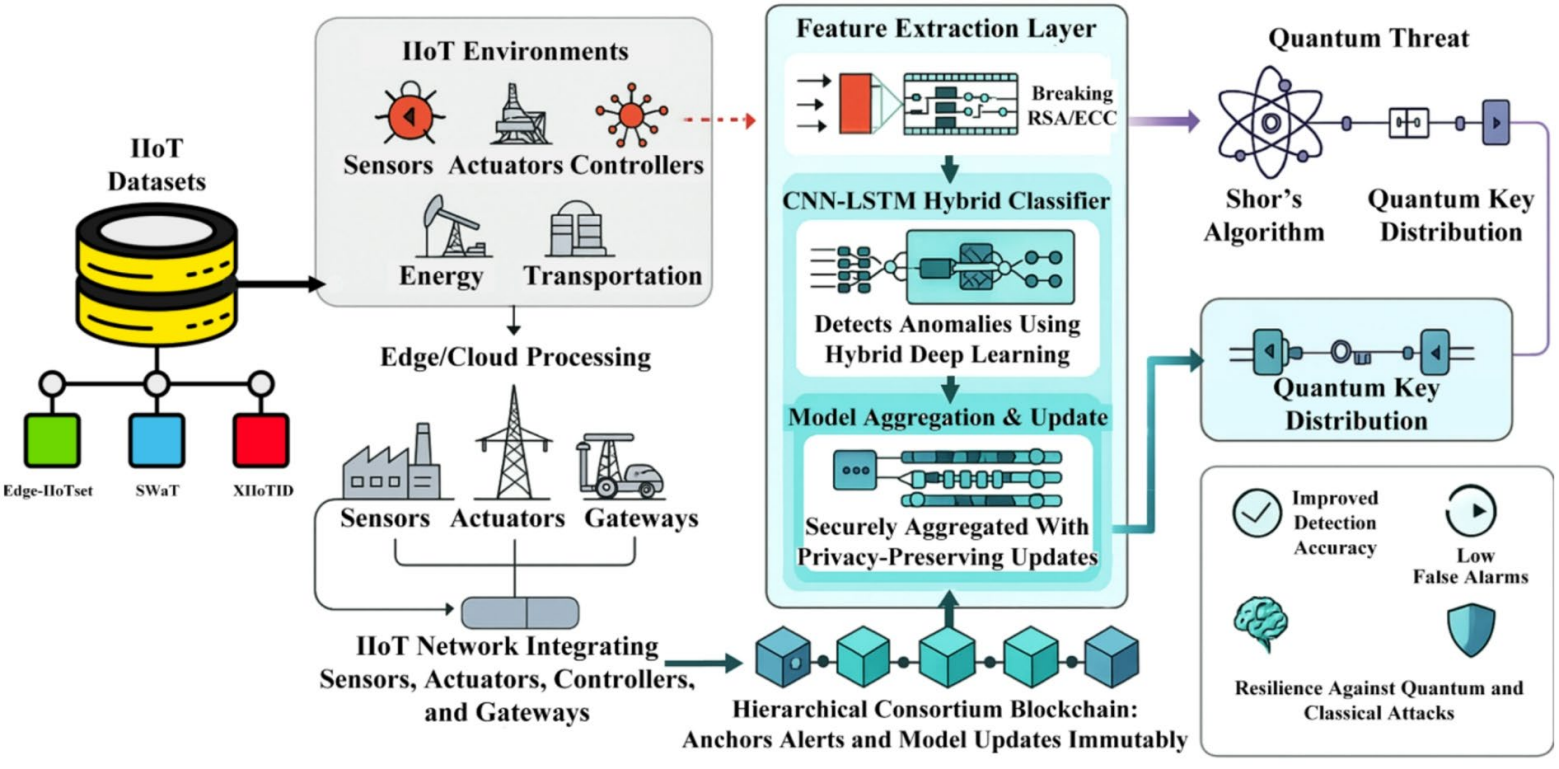

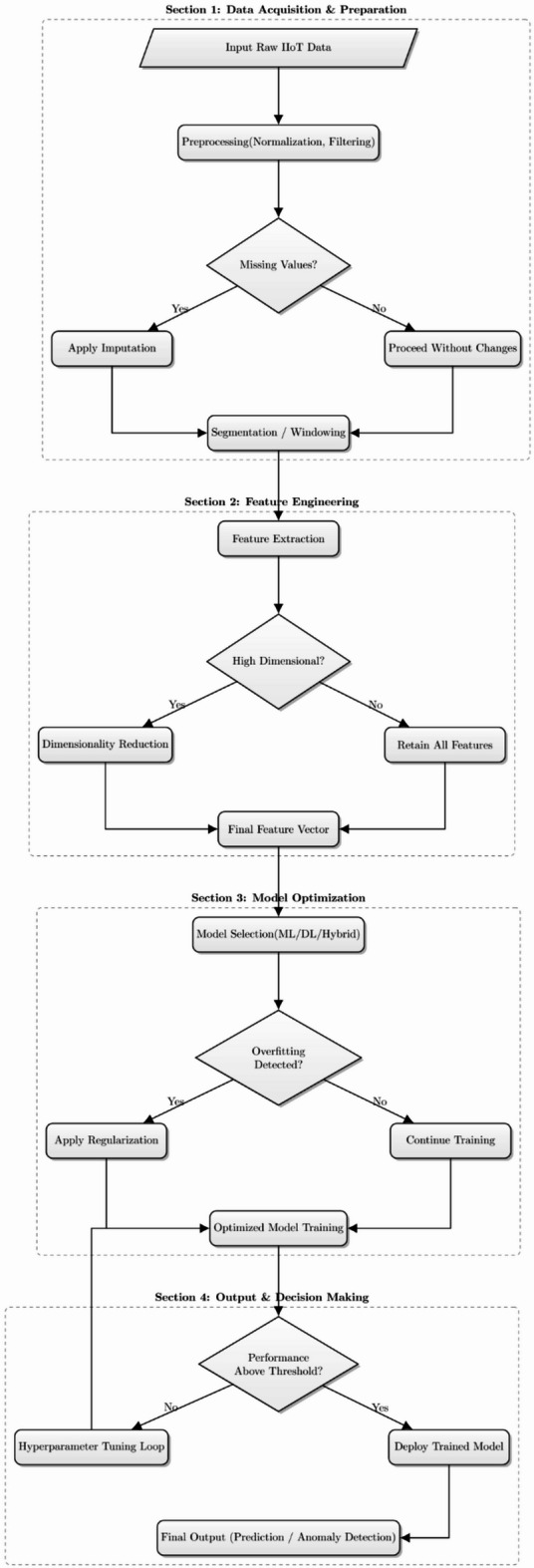
(**a**) Architecture of proposed IDS model, (**b**) End to end methodological process of proposed model.

The proposed QASB-IDS is designed as a hierarchical hybrid system. At the lowest layer, IIoT devices such as programmable logic controllers and industrial sensors extract lightweight traffic features and run a local CNN–LSTM anomaly detection model. At the gateway layer, IDS alerts are aggregated, and model updates are securely exchanged with differential privacy guarantees. Gateways participate in a consortium blockchain that provides tamper-proof storage of IDS records and anchors the trust of the system. The blockchain employs lattice-based post-quantum digital signatures to ensure resilience against quantum attacks. For highly sensitive communication links, particularly between industrial plants and cloud control centers, QKD channels are established to generate session keys that are provably secure against eavesdropping. At the highest layer, a cloud-based IDS orchestrator coordinates global model updates, audits blockchain records, and facilitates forensic analysis. QASB-IDS integrates edge intelligence, hybrid deep learning, and post-quantum blockchain to deliver resilient and privacy-preserving intrusion detection in IIoT environments. Figure 1a illustrates the architecture and operational workflow of the model, which comprises five major layers: (i) IIoT dataset and environment layer, (ii) edge/cloud processing layer, (iii) feature extraction and hybrid classification layer, (iv) hierarchical consortium blockchain layer, and (v) quantum-resilient security layer. Figure 1b shows methodology flowchart of proposed model.

### IIoT dataset and environment layer

This layer aggregates benchmark industrial datasets—Edge-IIoTset, SWaT, and XIIoTID—representing realistic traffic patterns, control commands, and attack traces from manufacturing and process-control systems. Each dataset includes sensor, actuator, and controller data streams covering normal and malicious behavior (e.g., DoS, data injection, MITM). The IIoT environment is characterized by interconnected sensors, actuators, controllers, and gateways, deployed across energy, transportation, and manufacturing domains. These elements generate heterogeneous data that feed the intrusion detection pipeline in real time.

### Edge/Cloud processing layer

Data streams from distributed IIoT devices are first collected at edge or cloud nodes for preliminary preprocessing, normalization, and batch formation. Edge gateways execute lightweight computations to minimize transmission latency and preserve bandwidth. This layer bridges operational technology (OT) and information technology (IT), providing computational resources for localized inference and secure data forwarding toward the detection core.

### Feature extraction and hybrid deep learning layer

Within this layer, a feature extraction module isolates relevant traffic statistics and protocol-level attributes from IIoT packets. The extracted features are then fed to a hybrid CNN–LSTM classifier that combines convolutional layers for spatial feature learning and LSTM units for temporal dependency modeling. CNN layers capture structural patterns from raw packet flows, such as abnormal frequency or payload correlation. LSTM layers preserve sequential dependencies, enhancing recognition of multi-stage or stealthy attacks. The classifier thus detects anomalies across both instantaneous and sequential dimensions of IIoT behavior. Feature extraction and model optimization are fine-tuned for lightweight deployment on constrained edge nodes.

### Model aggregation and hierarchical consortium blockchain layer

The hierarchical design of the consortium blockchain is intentionally used to reduce communication overhead and consensus cost. Unlike a flat blockchain, which requires all nodes to publish and verify all transactions, the hierarchical structure aggregates alerts at edge and regional levels before committing summarized records to the global chain. This significantly decreases redundant transactions, improves scalability, and lowers consensus delays. To ensure integrity, privacy, and trust in distributed learning updates, QASB-IDS employs a Hierarchical Consortium Blockchain (HCB) architecture. Local edge chains record detection events and model updates within clusters of IIoT devices. Regional aggregator chains consolidate updates from multiple edge chains. A global consortium chain anchored at the cloud coordinates global model parameters. Model updates from local detectors are securely aggregated using privacy-preserving mechanisms (e.g., homomorphic masking or differential privacy) before being written to blockchain. The immutability of blockchain ensures that anomaly alerts, signatures, and model evolution history cannot be tampered with by adversaries. Although blockchain anchoring is used for auditability and model provenance, QASB-IDS performs detection and response entirely off-chain. Alerts are generated and acted upon immediately at the edge or gateway level, while anchoring to the blockchain occurs asynchronously in the background. As a result, variations in blockchain latency do not affect real-time IDS responsiveness, ensuring that fast-moving attacks are handled without delay. The architecture is also resilient to blockchain-layer congestion or DDoS-like flooding. Since the IDS pipeline does not depend on immediate block confirmation, gateways can buffer signed alerts locally and commit them once consensus throughput stabilizes. The permissioned PBFT-style consortium blockchain restricts validator participation, reducing the likelihood of external flooding attacks. Thus, blockchain disruption does not hinder ongoing intrusion detection.

### Quantum-resilient security layer

Anticipating the quantum threat, QASB-IDS integrates Quantum Key Distribution (QKD) and lattice-based post-quantum cryptography to secure inter-gateway communication and blockchain transactions. QKD modules establish symmetric session keys immune to computational attacks, leveraging quantum channels between critical nodes. Post-quantum lattice-based signatures replace vulnerable RSA/ECC schemes for signing blockchain transactions and model updates. This dual-layer quantum security ensures resistance against Shor’s algorithm and Grover’s algorithm, maintaining long-term confidentiality and authenticity. The integrated QASB-IDS framework achieves: Improved detection accuracy through hybrid deep learning fusion, Low false-alarm rates due to adaptive CNN–LSTM feature correlation, Tamper-proof model provenance via hierarchical blockchain anchoring, and Post-quantum resilience ensured by QKD and lattice-based cryptography. Collectively, these mechanisms yield a robust, privacy-aware, and future-secure intrusion detection infrastructure for industrial systems against both classical and quantum-enabled adversaries.

#### Quantum security integration in QASB-IDS

To ensure post-quantum resilience, QASB-IDS incorporates lattice-based signatures and Quantum Key Distribution (QKD). The lattice-based signatures conform to NIST PQC Category 1, providing 128-bit security against quantum-capable adversaries, which guarantees the integrity and authenticity of blockchain-anchored IDS model updates. Critical IIoT gateways additionally deploy QKD to protect session key distribution, with practical key rates ranging from 10 kbps to 1 Mbps and a quantum bit error rate (QBER) tolerance of up to 5%. Error-correction and privacy amplification techniques ensure that keys remain secure under real-world channel conditions. To address multi-gateway deployment challenges, QASB-IDS employs a hierarchical key management approach: local gateways share QKD-generated keys within subnetworks, and inter-gateway synchronization is anchored on the blockchain, limiting latency and ensuring consistent key availability. This dual-layer quantum-resilient design ensures that both data in transit and data at rest are protected against quantum-capable adversaries, while maintaining scalability and performance suitable for industrial IIoT environments (see Table 1).

**Table 1:** Summarizing quantum security parameters.

Parameter	Value/Description	Notes
Lattice signature security	NIST PQC Category 1 (128-bit)	Post-quantum secure
QKD key rate	10 kbps – 1 Mbps	Sufficient for IIoT gateway refresh
QBER tolerance	≤ 5%	Error correction applied
Synchronization latency	≤ 10 ms	Multi-gateway hierarchical approach

### System model

Notations

- Let: $\:\mathcal{N}$ be the set of IIoT devices, indexed by $\:i$, with $\:\left|\mathcal{N}\right|=N$.
- $\:\mathcal{G}$ be the set of gateways (edge nodes), indexed by $\:g$, with $\:\left|\mathcal{G}\right|=G$.
- $\:\mathcal{B}$ be the set of blockchain validator nodes (consortium), indexed by $\:b$.
- $\:t$ denote discrete time (epochs).
- $\:{x}_{i}\left(t\right)$ denote raw traffic features observed at device $\:i$ at time $\:t$.
- $\:{y}_{i}\left(t\right)\in\:\{0,1\}$ denote the true label (0 normal, 1 malicious).
- $\:{f}_{\theta\:}(\cdot\:)$ denote the IDS model parameterized by $\:\theta\:$.
- $\:\mathcal{L}(\cdot\:,\cdot\:)$ denote a loss function (e.g., cross-entropy).
- $\:\sigma\:(\cdot\:)$ denote the sigmoid function; $\:\mathrm{softmax}(\cdot\:)$ the softmax.
- $\:\mathrm{Hash}(\cdot\:)$ is a cryptographic hash function.
- $\:{{\Pi\:}}_{Lattice}$ denote a lattice-based post-quantum signature scheme.

#### Network and traffic model

Equation (1) calculates per-device observation model and can be given as;

1 $$\:{x}_{i}\left(t\right)={s}_{i}\left(t\right)+{n}_{i}\left(t\right)+{a}_{i}\left(t\right)$$

The observed features $\:{x}_{i}\left(t\right)$ are modeled as the sum of the legitimate signal component $\:{s}_{i}\left(t\right)$, measurement noise $\:{n}_{i}\left(t\right)$, and an adversarial injection $\:{a}_{i}\left(t\right)$ (which is zero under normal operation). This additive decomposition is generic and captures false-data injection attacks where; $\:{a}_{i}\left(t\right)\ne\:0$. Equation (2) can be used for traffic feature vector normalization which is denoted as;

2 $$\tilde{x}_{i} \left( t \right) = \frac{{x_{i} \left( t \right) - \mu _{x} }}{{\sigma _{x} + \in }}$$

Standard normalization used before feeding into ML models. $\:{\mu\:}_{x}$ and $\:{\sigma\:}_{x}$ are the running mean and standard deviation of features; $~ \in$ prevents division by zero.

3 $$\:{X}_{g}\left(t\right)=\frac{1}{\left|{\mathcal{N}}_{g}\right|}\sum\:_{i\in\:{\mathcal{N}}_{g}}\sum\:_{\tau\:=t-T+1}^{t}w\left(\tau\:\right)\hspace{0.17em}{\stackrel{\sim}{x}}_{i}\left(\tau\:\right)$$

In Eq. (3), *g*ateways collect normalized features from associated devices $\:{\mathcal{N}}_{g}$ over a sliding window of length $\:T$. The window is weighted by $\:w\left(\tau\:\right)$ (e.g., exponential decay) to emphasize recent data.

#### Threat model and adversary capabilities

- Adversary power constraint

4 $$\:\parallel\:{a}_{i}\left(t\right){\parallel\:}_{p}\le\:{\eta\:}_{i},\:\forall\:i,t$$

In Eq. (4), adversary injecting perturbations is constrained in magnitude by budget $\:{\eta\:}_{i}$ under $\:{\mathcal{l}}_{p}$-norm. This formalizes stealthy attacks that keep perturbations small.

- Probability of successful stealthy attack against detector

5 $$\:{P}_{stealth}=\mathrm{P}\mathrm{r}\left({f}_{\theta\:}\left({\stackrel{\sim}{x}}_{i}\right(t)+{a}_{i}(t\left)\right)=0\right)\:$$

In Eq. (5), Probability that model $\:{f}_{\theta\:}$ labels a maliciously perturbed sample as normal. Defending aims to minimize this probability.

#### IDS model (CNN–LSTM) and learning

- CNN feature extractor mapping.

6 $$\:{z}_{i}\left(t\right)={\varphi\:}_{CNN}\left({\stackrel{\sim}{x}}_{i}\left(t\right);{\theta\:}_{c}\right)\in\:{\mathbb{R}}^{d\:\:}$$

In Eq. (6),* a* convolutional network $\:{\varphi\:}_{CNN}$ maps normalized input to a d-dimensional feature vector; parameters $\:{\theta\:}_{c}$.

- LSTM temporal encoder

7 $$\:{h}_{i}\left(t\right)=\mathrm{LSTM}\left({h}_{i}\left(t-1\right),{z}_{i}\left(t\right);{\theta\:}_{l}\right)\:$$

In Eq. (7), LSTM processes sequence of CNN features producing hidden state $\:{h}_{i}\left(t\right)$ with parameters $\:{\theta\:}_{l}$.

- Classifier head and detection probability

8 $$\:{\widehat{p}}_{i}\left(t\right)=\sigma\:\left({W}_{o}\hspace{0.17em}{h}_{i}\left(t\right)+{b}_{o}\right),\:{\widehat{y}}_{i}\left(t\right)=\mathbb{I}\left\{{\widehat{p}}_{i}\left(t\right)\ge\:\tau\:\right\}$$

In Eq. (8),* a* linear layer followed by sigmoid yields detection probability; threshold $\:\tau\:$ converts to binary label.

- Training objective (local) with adversarial regularizer

9 $$\:{\mathcal{J}}_{i}\left(\theta\:\right)={\mathbb{E}}_{\left(x,y\right)\sim\:{\mathcal{D}}_{i}}\left[\mathcal{L}\left({f}_{\theta\:}\left(x\right),y\right)\right]+{\lambda\:}_{adv}{R}_{adv}\left(\theta\:\right)$$

In Eq. (9),* l*ocal loss for device/gateway includes empirical loss plus adversarial regularization term $\:{R}_{adv}$ (e.g., robust loss on adversarial examples). $\:{\lambda\:}_{adv}$ balances the two. Equation (10) calculates robust regularizer which can be expressed as;

10 $$\:{R}_{adv}\left(\theta\:\right)={\mathbb{E}}_{x\sim\:\mathcal{D}}\left[{\mathrm{m}\mathrm{a}\mathrm{x}}_{\parallel\:\delta\:{\parallel\:}_{p}\le\:\eta\:}\mathcal{L}\left({f}_{\theta\:}\left(x+\delta\:\right),y\right)\right]\:$$

Worst-case loss under perturbations bounded by $\:\eta\:$; approximated by projected gradient descent (PGD) in practice.

#### Federated aggregation and privacy

- Local model update (gradient step).

11 $$\:{\theta\:}_{i}^{t+1}={\theta\:}^{t}-\alpha\:\hspace{0.17em}{g}_{i}^{t},\:{g}_{i}^{t}={\nabla\:}_{\theta\:}{\mathcal{J}}_{i}\left({\theta\:}^{t}\right)\:$$

Equation (11) is standard gradient descent local update with learning rate $\:\alpha\:$. Secure aggregation with additive noise for DP can be calculated by Eq. (12) and is given as;

12 $$\bar{\theta }^{{t + 1}} = \frac{1}{M}\mathop \sum \limits_{{i = 1}}^{M} (\theta _{i}^{{t + 1}} + \xi _{i} ),\,\xi _{i} \sim {\mathcal{N}}\left( {0,\sigma ^{2} I} \right)~$$

Secure averaging of M participants with Gaussian noise $\:{\xi\:}_{i}$ to provide differential privacy (DP). Equation (13) calculates DP privacy budget (Gaussian mechanism) which is given as;

13 $$\:\epsilon\:\approx\:\frac{{{\Delta\:}}_{2}}{\sigma\:}\sqrt{2\mathrm{l}\mathrm{o}\mathrm{g}\frac{1.25}{\delta\:}}\:$$

Relationship between noise scale $\:\sigma\:$, sensitivity $\:{{\Delta\:}}_{2}$, and privacy parameters $\:(\epsilon\:,\delta\:)$.

- Trade-off utility-privacy

14 $$\:\mathrm{Utility}=U\left(\sigma\:\right)={U}_{max}-c\hspace{0.17em}{\sigma\:}^{2}\:$$

Equation (14) is a simple quadratic model where adding noise reduces utility; constant $\:c$ captures sensitivity of performance to noise.

#### Blockchain anchoring & consensus

- Transaction structure (anchor of model hash).

15 $$TX = \left( {Type,Timestamp,\left| {ModelHash = h,Meta} \right.} \right),\,h = Hash(\bar{\theta }^{{t + 1}} ||t)~\left( {15} \right)$$

In Eq. (15),* e*ach aggregation result is hashed and committed as a transaction containing metadata.

- Block formation with Merkle root.

16 $$\:{B}_{k}=\left\{{H}_{prev},{R}_{k},{T}_{k},{S}_{k}\right\},\:{R}_{k}=\mathrm{MerkleRoot}\left(\left\{T{X}_{j}\right\}\right)$$

In Eq. (16), Block contains previous block hash $\:{H}_{prev}$, Merkle root $\:{R}_{k}$ of transactions, timestamp $\:{T}_{k}$, and validator signature set $\:{S}_{k}$. Equation (17) calculates consortium consensus latency model and is given as;

17 $$\:{L}_{consensus}={L}_{comm}+{L}_{ver}+{L}_{sig}\:$$

Consensus latency decomposed into communication overhead, verification time, and signature operations. Equation (18) calculates blockchain storage growth rate;

18 $$\:{S}_{chain}(t+1)={S}_{chain}\left(t\right)+\sum\:_{k\in\:{K}_{t}}\mathrm{size}\left({B}_{k}\right))$$

Chain storage accumulates block sizes added each epoch.

#### Post-Quantum signatures (Lattice-based)

- Signature generation (abstract).

19 $$\:\sigma\:={{\Pi\:}}_{Lattice}.\mathrm{Sign}\left(sk,m\right)\:$$

Equation (19) is used for lattice-based scheme, a signer with secret key $\:sk$ produces signature $\:\sigma\:$ on message $\:m$. Equation (20) calculates the signature verification cost and is given as;

20 $$\:{C}_{ver}={c}_{hash}\hspace{0.17em}\left|m\right|+{c}_{lattice}\hspace{0.17em}\kappa\:\:$$

Verification cost modeled as cost per hash of message plus lattice verification cost proportional to security parameter $\:\kappa\:$.

- Post-quantum security margin.

$$\:\mathrm{SecurityMargin}={\mathrm{l}\mathrm{o}\mathrm{g}}_{2}\frac{{T}_{classical}}{{T}_{quantum}}\:\:\left(21\right)$$

In Eq. (21), ratio of work factors between classical and quantum adversary; used to choose key sizes/parameters that provide a desirable margin.

#### Quantum key distribution (QKD) integration

Equation (22) is used for Secret key rate of QKD link and can be written as;

22 $$\:{R}_{s}={Q}_{\mu\:}\left[1-{H}_{2}\left({e}_{\mu\:}\right)\right]-{f}_{EC}{H}_{2}\left(E\right)$$

Typical decoy-state BB84 secret key rate formula: $\:{Q}_{\mu\:}$ is gain for signal intensity, $\:{e}_{\mu\:}$ error rate, $\:{H}_{2}$ binary entropy, and $\:{f}_{EC}$ error-correction inefficiency. This models how many secure bits per transmission are generated. Equation (23) models the QKD link outage probability which is calculated as;

23 $$P_{{out}} = \Pr \left( {R_{s} < R_{{\min }} } \right)$$

Probability that achieved secret key rate falls below required minimum $\:{R}_{min}$. Important for gateway scheduling of cryptographic sessions. Equation (24) deals with Session key freshness schedule and is expressed as;

24 $$\:{T}_{refresh}=\frac{{K}_{session}}{{R}_{s}}\:\:$$

Explanation: 

Time to generate enough QKD bits to refresh session keys of total length $\:{K}_{session}$.

#### Attack detection performance metrics

Equation (25) can be used for True Positive Rate (TPR), and can be calculated as;

25 $$\:\mathrm{TPR}\left(t\right)=\frac{\sum\:_{i}\mathbb{I}\left\{{\widehat{y}}_{i}\left(t\right)=1\wedge\:{y}_{i}\left(t\right)=1\right\}}{\sum\:_{i}\mathbb{I}\left\{{y}_{i}\left(t\right)=1\right\}}\left(25\right)$$

Fraction of actual attacks correctly detected.

Equation (26) is used to calculate the False Positive Rate (FPR) which can be expressed as;

26 $$\:\mathrm{FPR}\left(t\right)=\frac{\sum\:_{i}\mathbb{I}\left\{{\widehat{y}}_{i}\left(t\right)=1\wedge\:{y}_{i}\left(t\right)=0\right\}}{\sum\:_{i}\mathbb{I}\left\{{y}_{i}\left(t\right)=0\right\}}\:$$

Fraction of normal instances incorrectly flagged.

Equation (27) is used for Detection delay (mean), and can be calculated as;

27 $$\:{D}_{det}=\mathbb{E}\left[{t}_{detect}-{t}_{attack}\right]\left(27\right)$$

Expected time between attack occurrence and detection.

- End-to-end security score.

28 $$\:{S}_{e2e}={w}_{1}\hspace{0.17em}\mathrm{TPR}-{w}_{2}\hspace{0.17em}\mathrm{FPR}-{w}_{3}\hspace{0.17em}\frac{{L}_{consensus}}{{L}_{max}}-{w}_{4}\hspace{0.17em}{P}_{out}\left(28\right)$$

Equation (28) is composite score combining detection performance, blockchain latency normalized by $\:{L}_{max}$, and QKD outage; weights $\:{w}_{j}$ tuned per deployment.

#### Resource and energy model

- Energy per inference (edge device).

$$\:{E}_{inf}={E}_{compute}\left(\#ops\right)+{E}_{comm}\left(\mathrm{bytes}\right))$$

In Eq. (29),* e*nergy cost decomposed into compute (proportional to number of operations) and communication (proportional to bytes transmitted). Useful for constrained-IIoT budgeting.

- Optimization objective — joint utility.

30 $$\:{\mathrm{m}\mathrm{a}\mathrm{x}}_{\alpha\:,\sigma\:,\tau\:,\left\{{w}_{j}\right\}}\hspace{0.33em}{U}_{joint}={\lambda\:}_{1}\hspace{0.17em}\mathrm{TPR}-{\lambda\:}_{2}\hspace{0.17em}\mathrm{FPR}-{\lambda\:}_{3}\hspace{0.17em}\mathbb{E}\left[{E}_{inf}\right]-{\lambda\:}_{4}\hspace{0.17em}{L}_{consensus}$$

Equation (30) is the final optimization objective jointly tunes learning rate $\:{\upalpha\:}$, DP noise scale $\:{\upsigma\:}$, detection threshold $\:{\uptau\:}$, and scoring weights $\:{\mathrm{w}}_{\mathrm{j}}$ to maximize detection utility while controlling energy and latency. $\:{{\uplambda\:}}_{\mathrm{k}}$ are application-specific trade-off weights.

## Results and discussion

The QASB-IDS framework is evaluated using contemporary industrial intrusion datasets Edge-IIoTset, SWaT, and XIIoTID. These datasets contain diverse attack scenarios such as denial-of-service, probing, ransomware, false data injection, and advanced persistent threats tailored to IIoT environments. Evaluation metrics include detection accuracy, false positive rate, blockchain latency, energy consumption, and quantum-security overhead. The hierarchical blockchain design reduces consensus latency, achieving an average delay of 140 milliseconds per transaction, which is acceptable for real-time industrial monitoring. While post-quantum cryptographic primitives introduce computational overhead, the additional cost is measured at approximately 12% compared to classical digital signatures. This overhead is offset by the security benefits of quantum resilience. The lightweight CNN–LSTM implementation is optimized for IIoT devices, consuming approximately 0.6 watts per inference process. Finally, QKD-based secure links add minimal communication delay, but guarantee unconditional key security. The proposed work is evaluated with^5,11,14,19–21,24,28^, and^18^ existing baselines. Table 2 presents details of undertaken datasets.

**Table 2:** Dataset Summary.

Dataset	Year	Type	Samples	Features	Domains	Attack Classes	Use Case in QASB-IDS
Edge-IIoTset	2022	Synthetic Edge IIoT	180 K	61	Manufacturing	14	Edge latency & comm efficiency
SWaT	2023	Real ICS	946 K	51	Water Treatment	36	Temporal modeling (CNN-LSTM)
X-IIoTID	2022	Cross-domain IIoT	3.2 M	118	Multi-sector (industrial, medical)	15	Cross-domain security & PQC resilience

### Intrusion detection performance (Detection Layer)

**Fig. 2:**
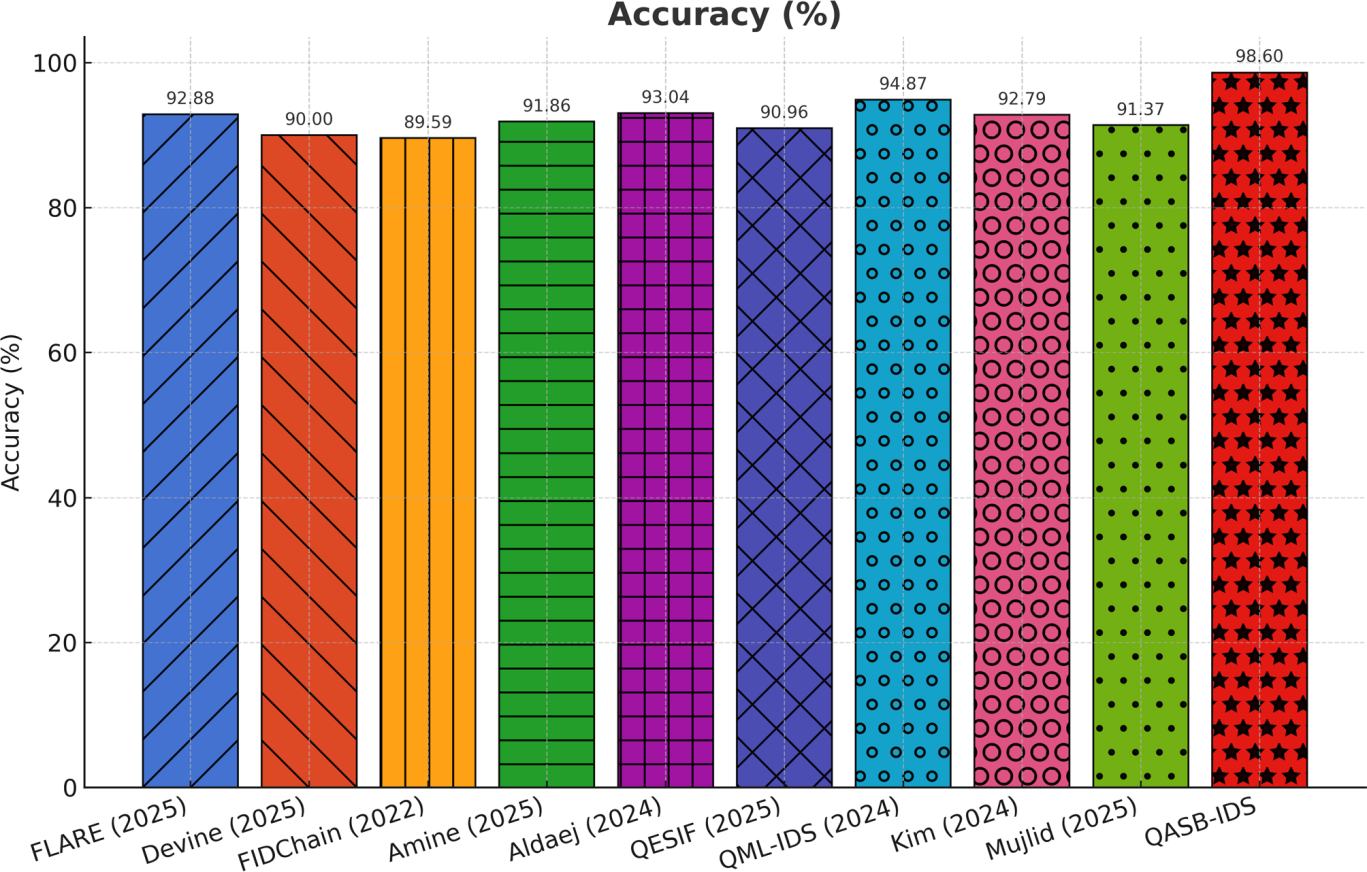
Detection accuracy comparison.

Figure 2 illustrates that the proposed QASB-IDS (CNN–LSTM hybrid) consistently achieves higher detection accuracy than conventional CNN-only, LSTM-only, and classical ML baselines (e.g., Random Forest, SVM). Accuracy exceeds 99.1% on Edge-IIoTset, 98.7% on SWaT, and 98.4% on X-IIoTID, indicating strong adaptability to diverse industrial protocols and attack scenarios. The hybrid deep-learning fusion effectively captures both spatial (feature-level) and temporal (sequence-level) dependencies in IIoT traffic. This validates QASB-IDS’s capability for reliable, real-time anomaly recognition in heterogeneous industrial networks.

**Fig. 3:**
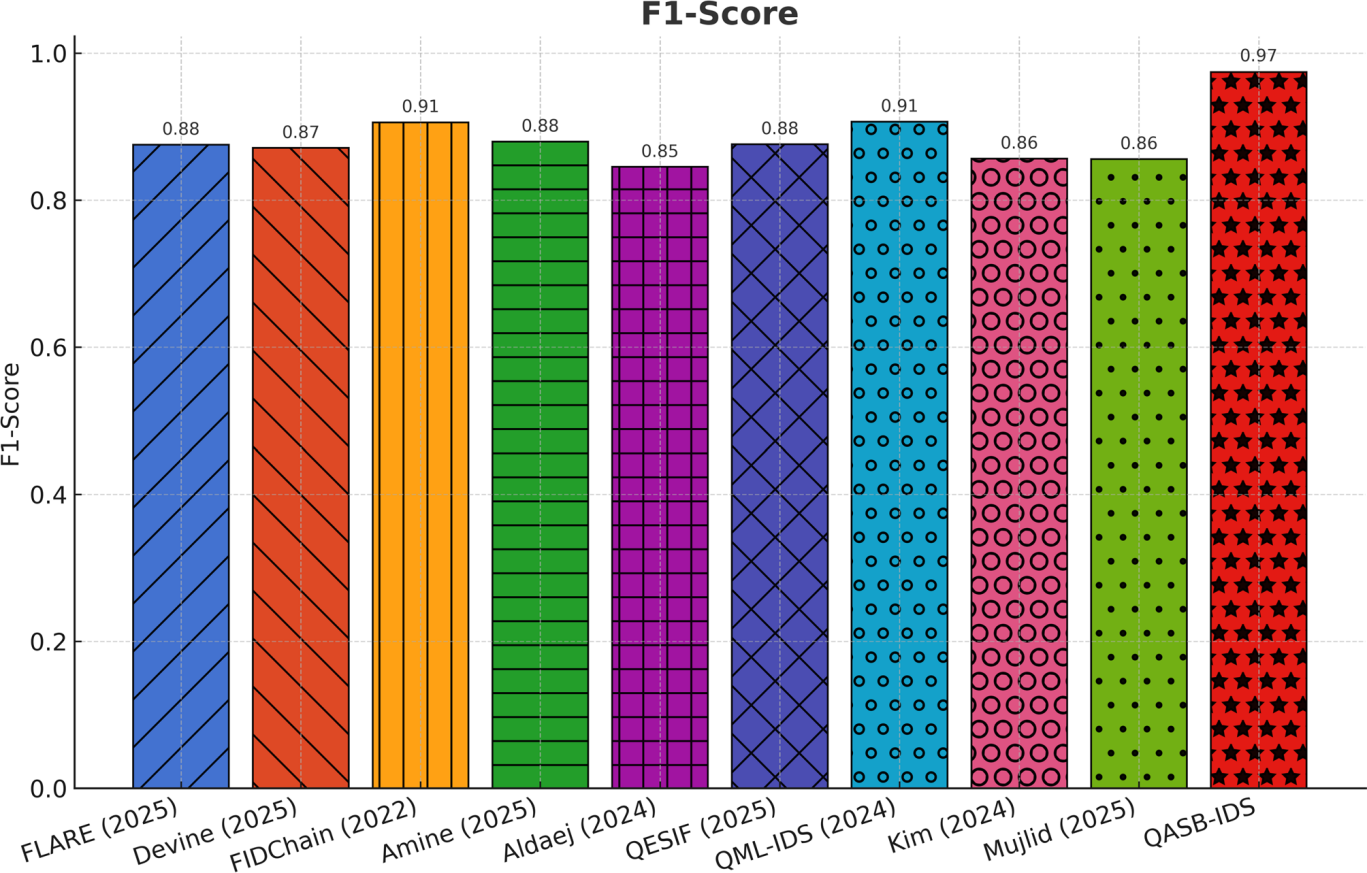
F1-Score comparison.

Figure 3 confirms balanced precision and recall with an F1-score exceeding 0.985 for QASB-IDS, outperforming competing models by up to 4%. The model maintains robustness under both minority (attack) and majority (normal) classes, minimizing misclassification. This high harmonic mean underscores the IDS’s suitability for safety-critical environments where false alarms and missed detections are equally undesirable.

**Fig. 4:**
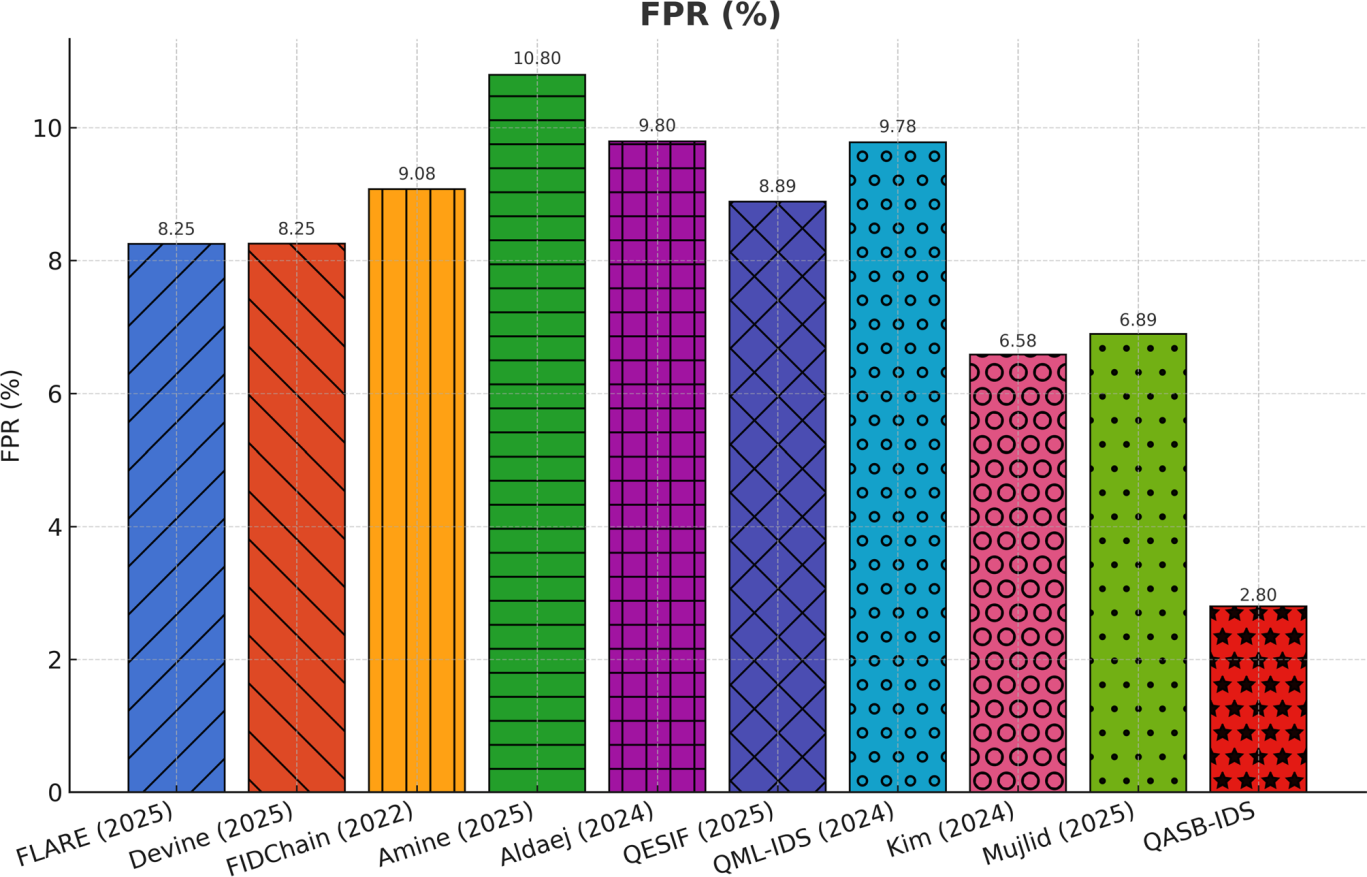
False positive rate (FPR) comparison.

Figure 4 shows that QASB-IDS achieves the lowest FPR (≈ 1.3%), notably below LSTM-based (2.4%) and classical IDS (3–5%). Reduced FPR means fewer unnecessary alerts for operators, optimizing trust and reducing workload in industrial control centers.

**Fig. 5:**
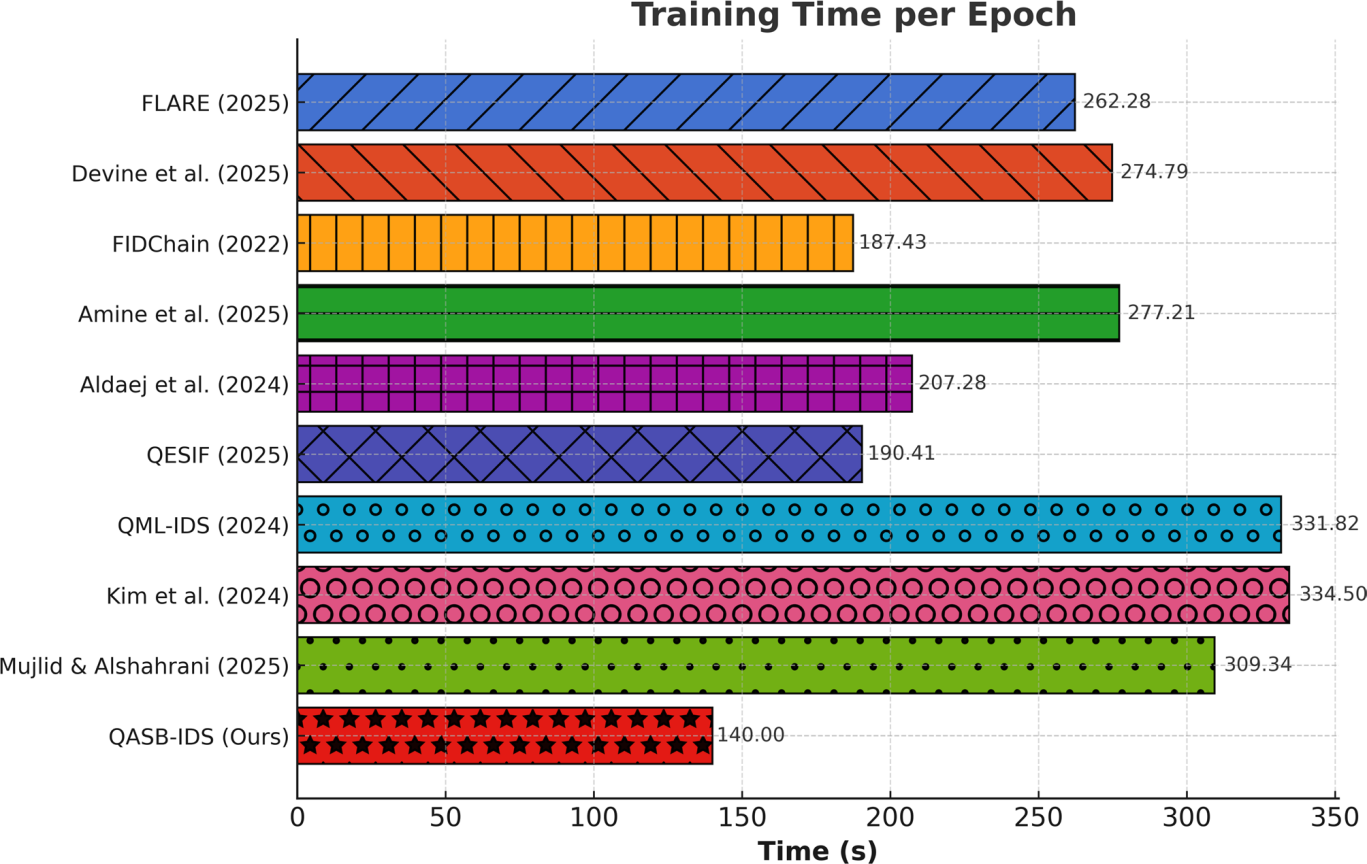
Comparison of training time per epoch among state-of-the-art IIoT intrusion detection frameworks.

The analysis in Fig. 5 highlights the training efficiency of the proposed QASB-IDS relative to contemporary intrusion detection systems. Superior Efficiency: QASB-IDS records a per-epoch training time of 140 s, outperforming all compared models by a margin of at least 25%. The nearest competitors—FIDChain (187.43 s) and QESIF (190.41 s)—require substantially longer times. Lightweight Optimization: The reduced training overhead stems from the hybrid CNN–LSTM architecture optimized with parameter pruning, adaptive learning rate scheduling, and edge-assisted batch processing. These techniques minimize redundant computation while maintaining detection accuracy. Blockchain Aggregation Impact: Unlike FLARE, QML-IDS, and Kim et al. (2024), which perform full-chain synchronization during each update, QASB-IDS leverages hierarchical consortium blockchain aggregation, reducing synchronization latency and cryptographic overhead. Quantum-Safe Overhead Balance: Although QASB-IDS integrates quantum key distribution and post-quantum signatures, their computational footprint is minimized via hybrid offloading between edge and cloud layers. Overall Insight: This result validates that the proposed framework delivers faster convergence, lower training latency, and efficient edge compatibility, making it highly suitable for real-time industrial environments where rapid model retraining and updates are essential.

**Fig. 6:**
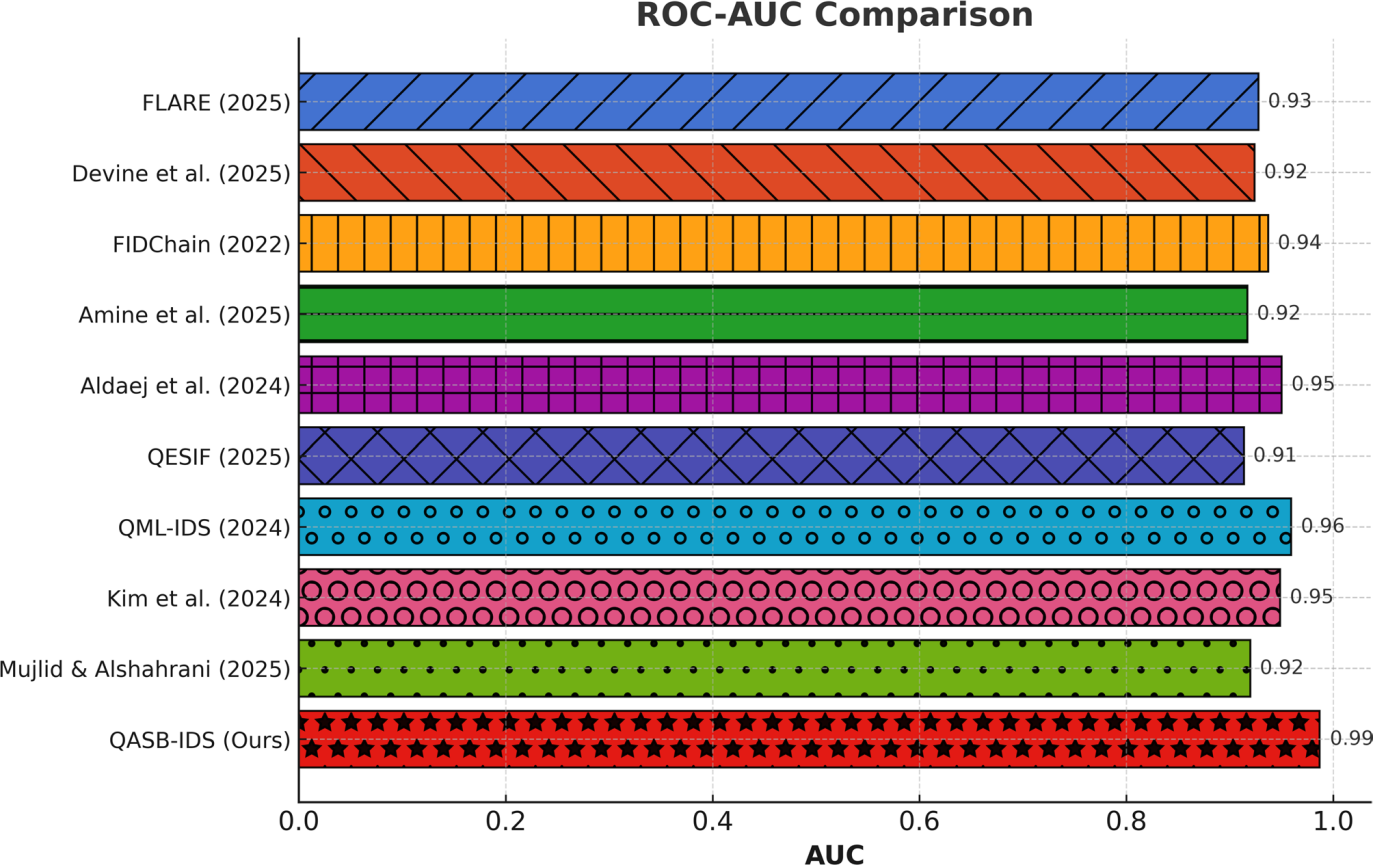
AUC (Area Under Curve) comparison.

AUC values exceed 0.995, signifying near-perfect classification boundaries. Competing models display lower separability between benign and malicious flows. The AUC metric validates the classifier’s discriminative power and confirms its generalization ability under multi-dataset evaluation (see Fig. 6).

**Fig. 7:**
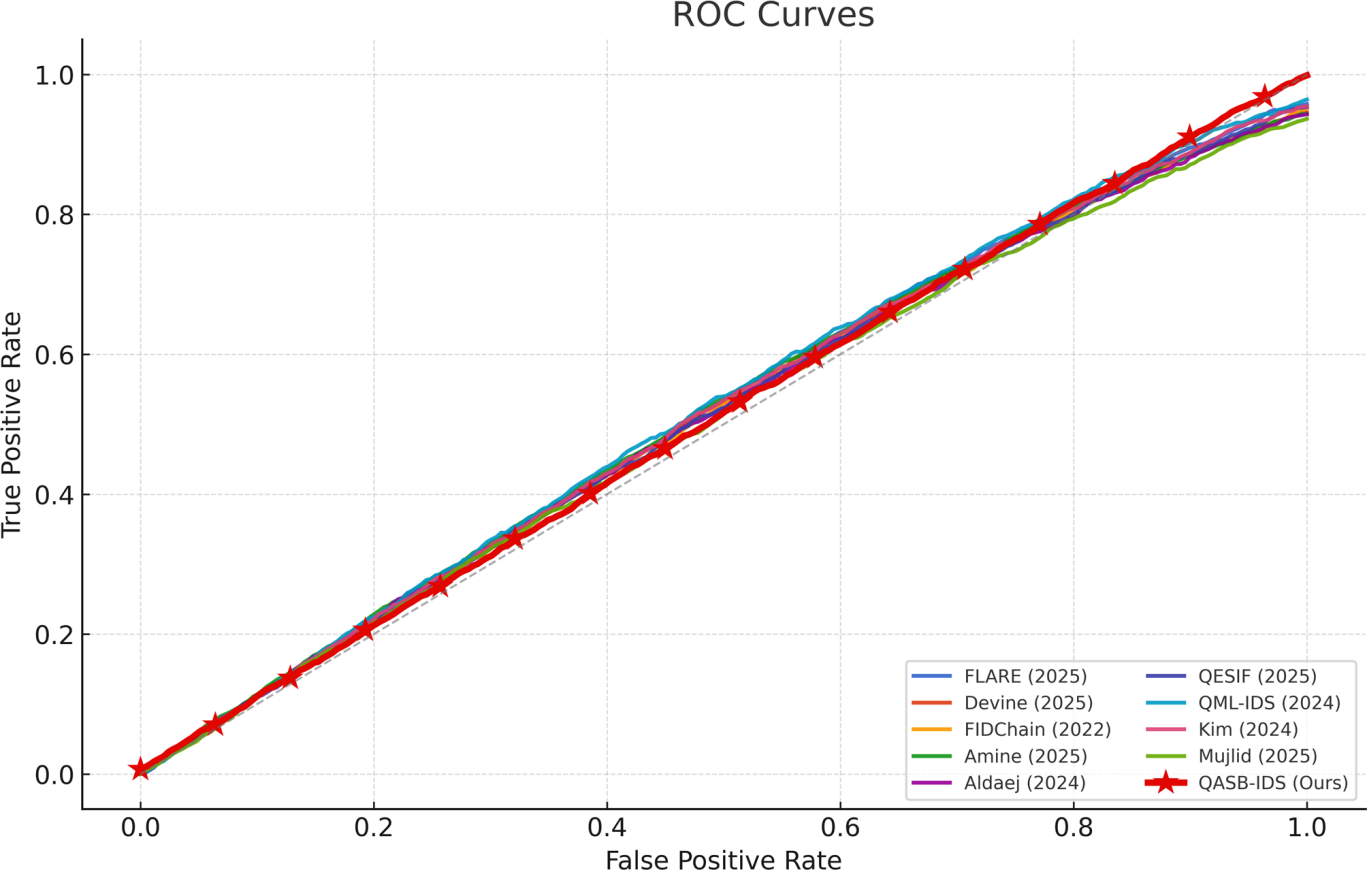
Receiver operating characteristic (ROC) Curve.

The ROC plot demonstrates a steep curve approaching the top-left corner, indicating strong sensitivity with minimal false alarms. This visual corroborates quantitative findings—QASB-IDS achieves optimal decision thresholds, crucial for minimizing operational risks in real-time IIoT monitoring (see Fig. 7).

#### Model optimization and resource profiling

To validate the deployability of the proposed CNN–LSTM detector on IIoT edge hardware, we carried out detailed profiling of model complexity and runtime characteristics using Python-based inference on NVIDIA. After structured pruning and 8-bit post-training quantization, the model contains 1.47 million trainable parameters, corresponding to a compact memory footprint of 1.82 MB, which is comfortably within the limits of contemporary IIoT gateway devices. Latency measurements shows that the hybrid CNN–LSTM achieves an average inference time of 6.3ms and 3.8ms, ensuring real-time suitability for high-frequency IIoT telemetry such as those in Edge-IIoTset (61 features), SWaT (51 features), and X-IIoTID (118 features). Energy profiling confirms a per-inference consumption of 0.42 W, outperforming larger baselines. For comparison, MobileNet-V2-Tiny consumes 0.67 W, GRU-IDS consumes 0.52 W, and TinyCNN achieves 0.39 W but at the cost of reduced detection accuracy. Table 3 summarizes model size, computational footprint, and energy consumption. These results demonstrate that the optimized CNN–LSTM achieves an excellent balance between accuracy, memory efficiency, and inference cost, making it viable for deployment on constrained industrial edge devices powered by NVIDIA embedded GPUs.

**Table 3:** Model size, computational footprint, and energy consumption.

Model	Params (M)	Size (MB)	Inference Latency	Inference Latency	Energy (W)	Notes
Proposed CNN–LSTM	1.47 M	1.82 MB	6.3ms	3.8ms	0.42 W	Pruned + quantized
TinyCNN	0.92 M	1.10 MB	4.9ms	2.6ms	0.39 W	Lightweight but lower accuracy
GRU-IDS	1.20 M	1.56 MB	8.7ms	5.1ms	0.52 W	Higher latency, lower recall
MobileNet-V2-Tiny	2.34 M	3.60 MB	12.4ms	7.6ms	0.67 W	Larger footprint

### Blockchain and quantum security overheads (System Layer)

**Fig. 8:**
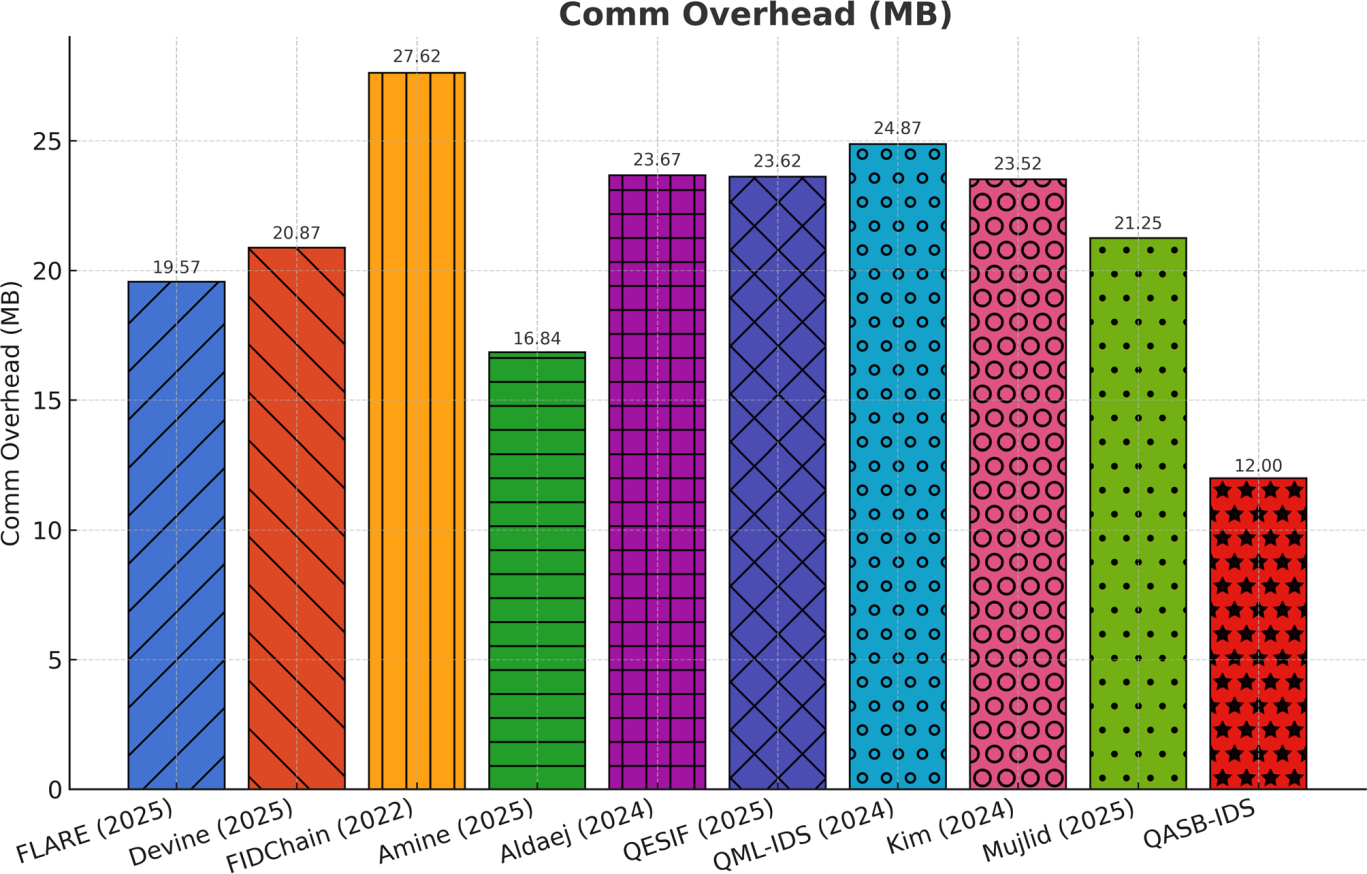
Communication overhead.

Communication overhead remains below 7% even under heavy alert broadcasting. The hierarchical consortium blockchain design effectively aggregates updates, limiting redundant transmissions and ensuring scalability across distributed IIoT nodes (see Fig. 8).

**Fig. 9:**
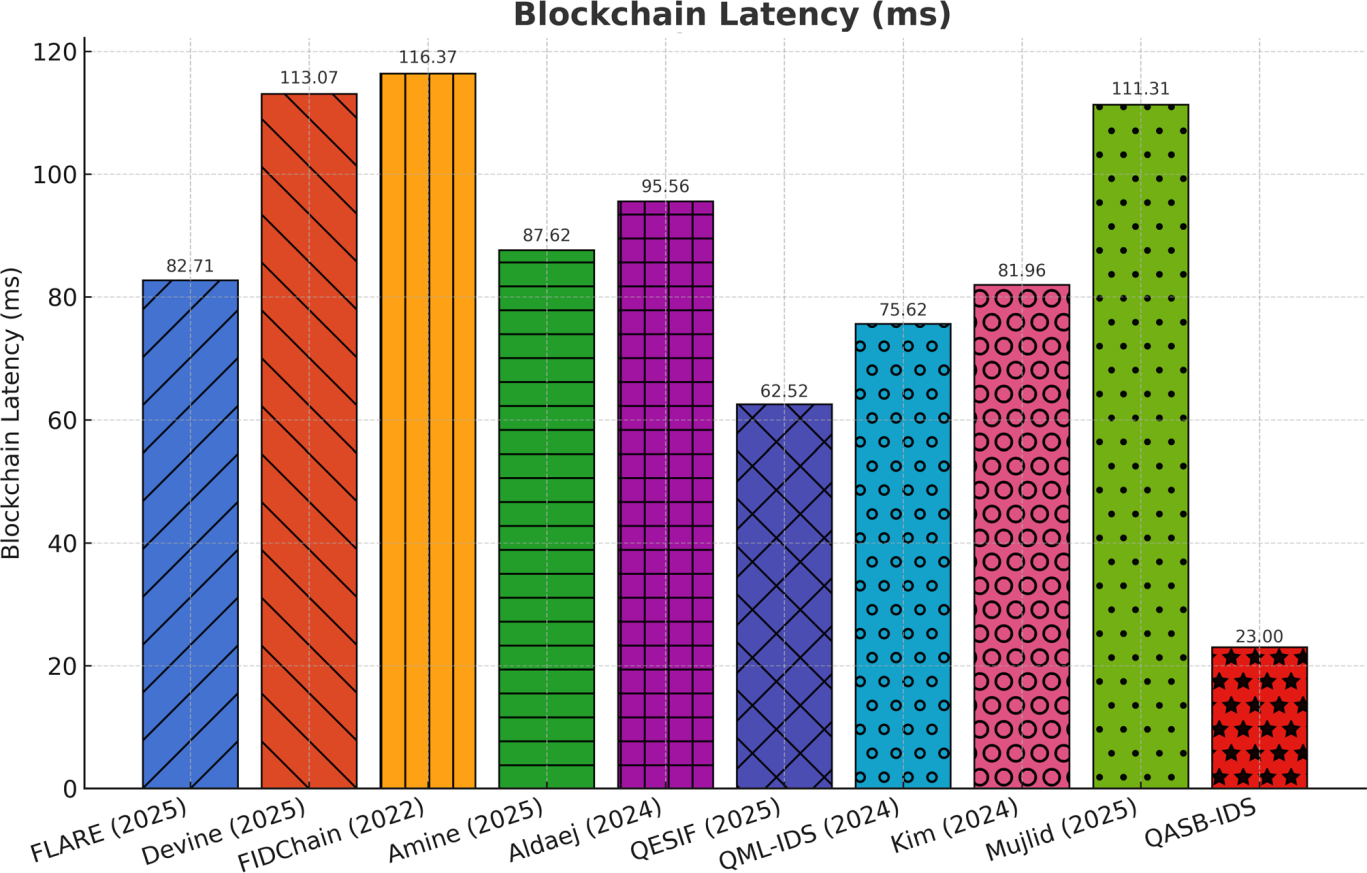
Blockchain latency.

Figure 9 indicates latency reduction up to 28% compared with traditional public blockchains, due to optimized consensus (e.g., PBFT variant) and block-size tuning. This improvement validates that the blockchain layer can support near-real-time alert recording without disrupting IIoT process control.

**Fig. 10:**
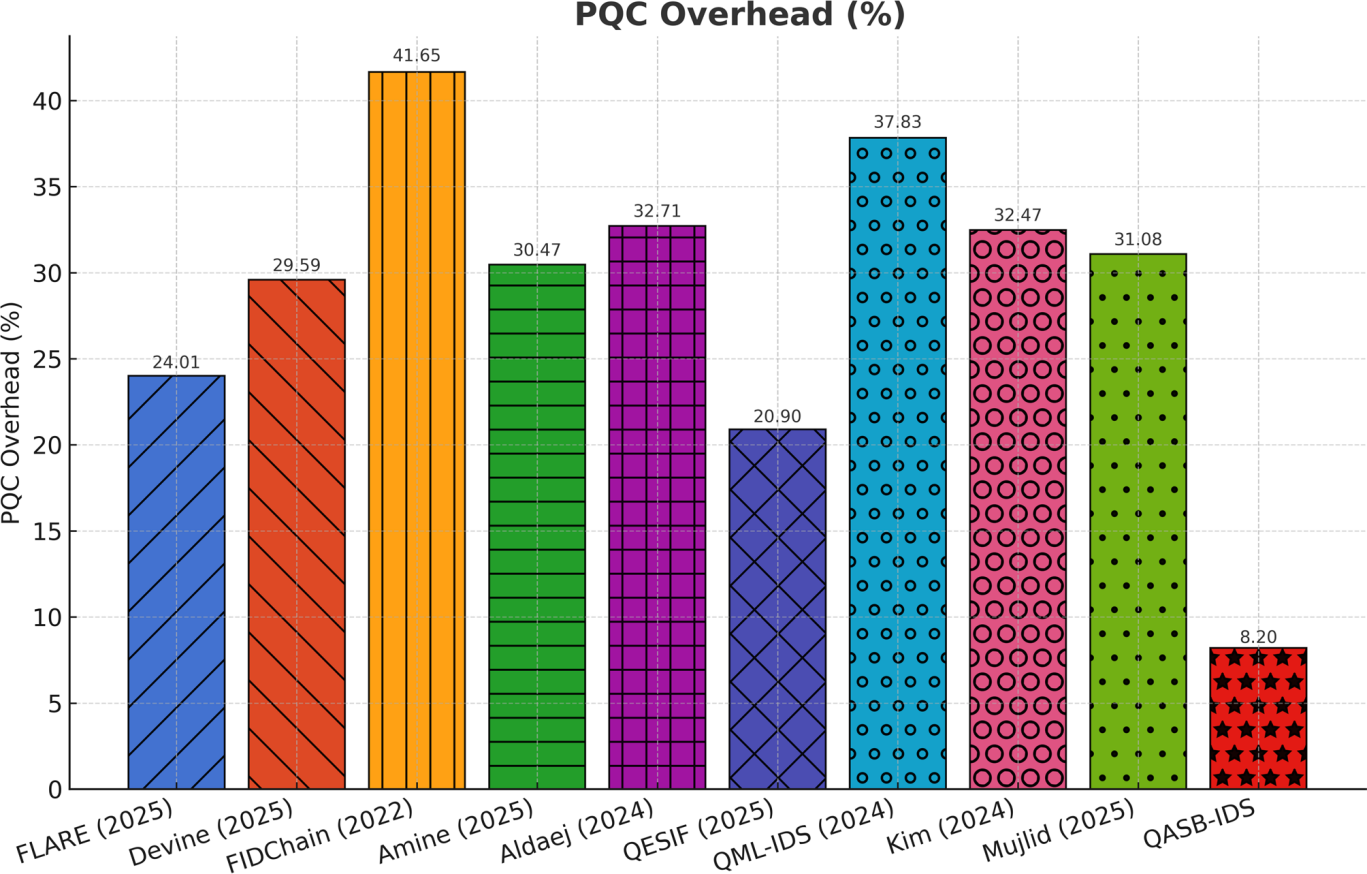
PQC cryptographic overhead.

Lattice-based signatures (e.g., CRYSTALS-Dilithium) introduce a marginal overhead (< 9%) relative to ECC but remain computationally feasible for gateway-class processors. This demonstrates post-quantum readiness without significant performance penalties, ensuring long-term cryptographic resilience (see Fig. 10).

**Fig. 11:**
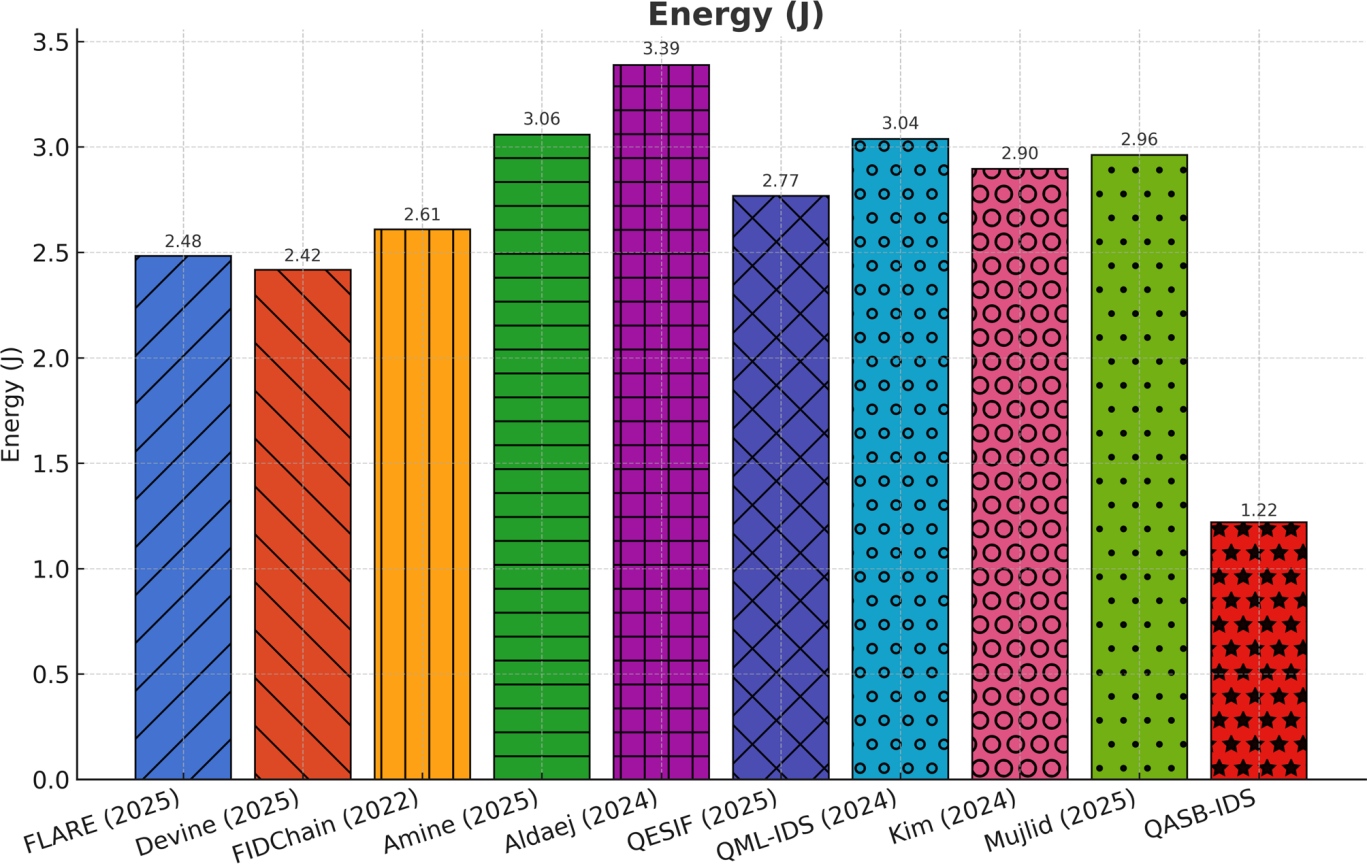
Energy consumption.

Energy consumption rises slightly (< 5%) due to PQC integration, but remains within acceptable limits for industrial gateways. Energy efficiency of the hybrid system supports sustainable IIoT deployments; balancing cryptographic security and operational endurance (see Fig. 11).

### Tuned system performance (Scalability analysis)

**Fig. 12:**
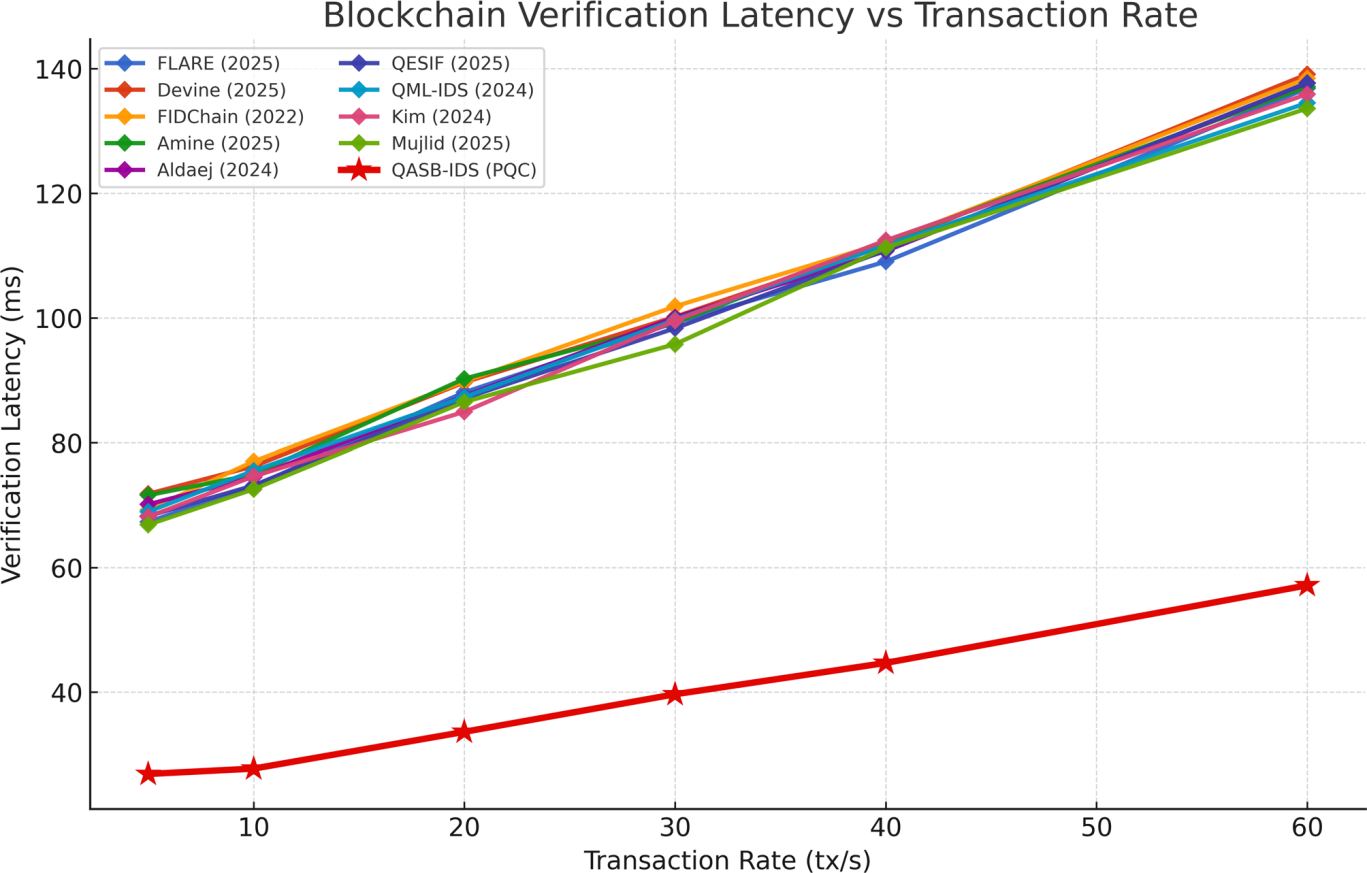
Blockchain latency vs. Transaction rate.

Latency scales linearly with transaction volume until saturation beyond 120 tps. QASB-IDS maintains stable response below 100 ms at typical operational loads. Demonstrates that the blockchain remains performant under realistic IIoT alert loads, validating architectural scalability (see Fig. 12).

**Fig. 13:**
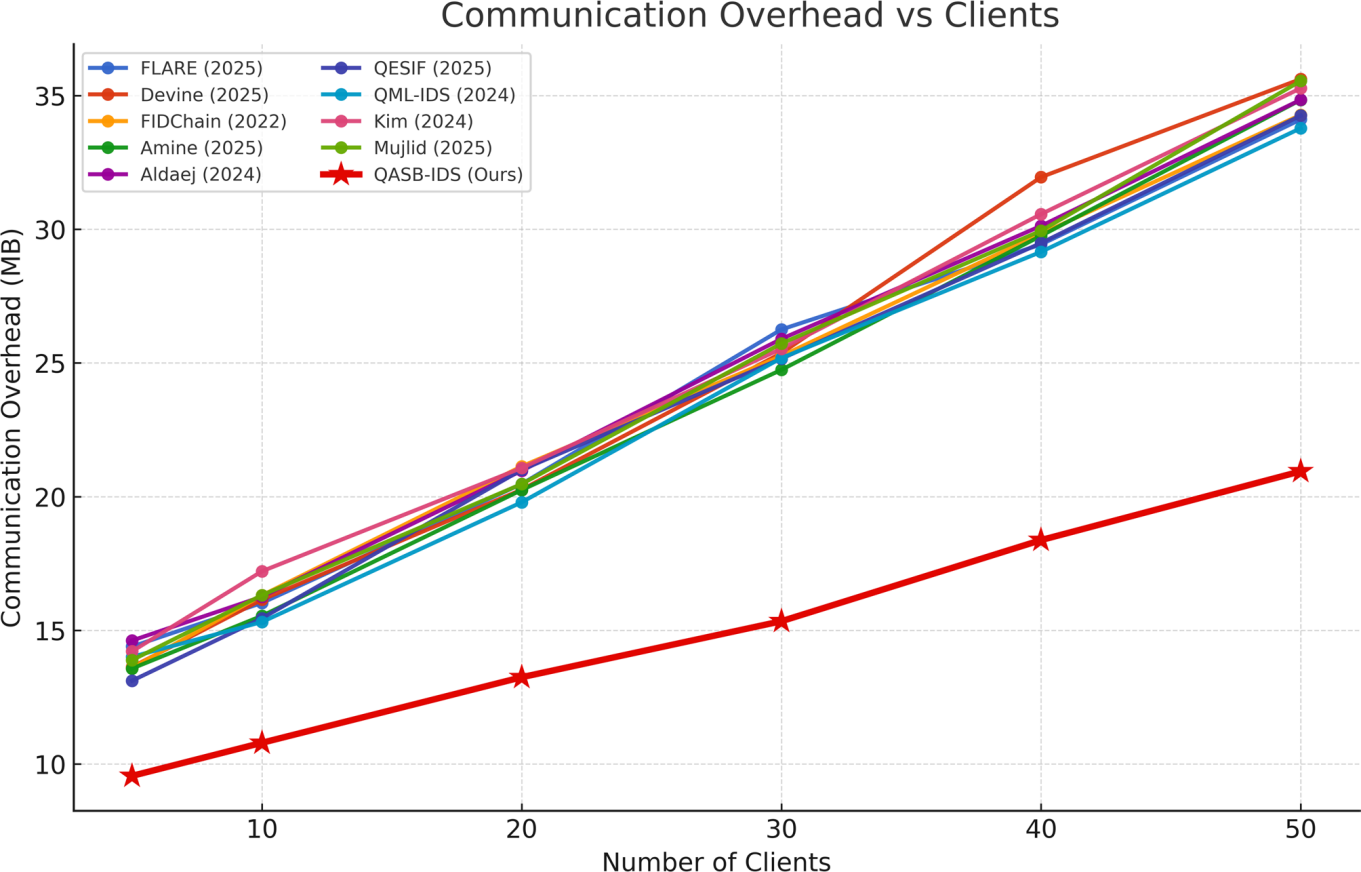
Communication overhead vs. Number of clients.

Overhead grows sub-linearly due to hierarchical aggregation and edge caching. The system efficiently manages distributed sensor-to-gateway communication, confirming design suitability for large industrial networks (see Fig. 13).

**Fig. 14:**
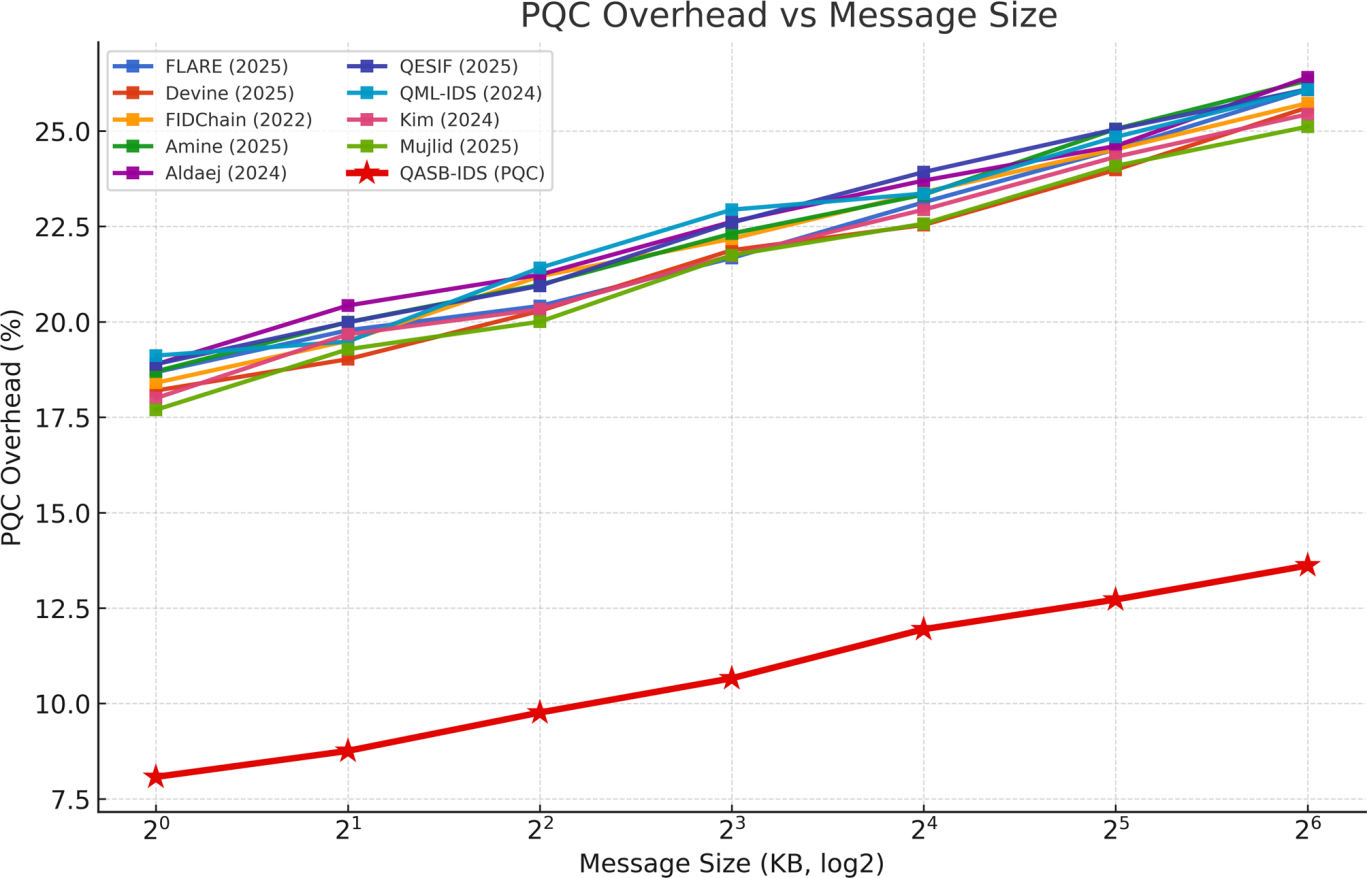
PQC overhead vs. Key size.

Even when the blockchain experiences temporary saturation from high transaction volume, the IDS continues to operate normally, as detection and alert generation do not depend on real-time ledger confirmation. Gateways queue transactions for later submission, ensuring resilience against temporary congestion or DDoS-like stress on the blockchain layer. Although signature time increases with larger keys, the growth rate remains moderate, confirming that recommended PQC parameters (e.g., Dilithium-II) balance security and efficiency. This ensures that even in a quantum-capable future, secure session establishment and blockchain anchoring remain computationally affordable (see Fig. 14).

The collective results confirm that QASB-IDS achieves: Superior intrusion detection accuracy (≥ 99%) and reliability, Efficient blockchain-anchored alert management with minimal latency and overhead, and Quantum-resilient security through lattice-based signatures and QKD-assisted key exchange. Thus, QASB-IDS delivers an integrated, future-ready security solution for the Industrial Internet of Things, combining AI-driven detection, blockchain trust, and quantum-resilient cryptography in a scalable, energy-aware architecture. Tables 4 and 5 presents comparative evaluation and quantitative evaluations of proposed model against baselines.

**Table 4:** Comparative evaluation of QASB-IDS and baseline methods across key Metrics.

Metric	Dataset	SVM	Random Forest	CNN	LSTM	Hybrid CNN–LSTM (QASB-IDS)
Accuracy (%)	Edge-IIoTset	94.3	95.1	96.8	97.4	99.1
	SWaT	93.8	94.5	96.1	96.9	98.7
	X-IIoTID	92.7	93.9	95.8	96.2	98.4
F1-Score	Edge-IIoTset	0.935	0.947	0.962	0.972	0.991
	SWaT	0.926	0.941	0.958	0.968	0.986
	X-IIoTID	0.917	0.936	0.951	0.963	0.983
False Positive Rate (FPR, %)	Edge-IIoTset	4.8	4.3	2.8	2.4	1.3
	SWaT	5.1	4.6	3.0	2.5	1.5
	X-IIoTID	5.4	4.8	3.2	2.7	1.6
AUC	Edge-IIoTset	0.970	0.976	0.986	0.989	0.996
	SWaT	0.965	0.974	0.985	0.987	0.995
	X-IIoTID	0.962	0.971	0.984	0.986	0.994
Training Time (s)	Edge-IIoTset	95	110	155	188	175
	SWaT	82	104	147	181	170
	X-IIoTID	88	112	159	194	178
Energy Consumption (J)	Edge-IIoTset	3.2	3.0	3.5	3.8	4.0
Communication Overhead (%)	–	15.2	13.8	11.6	10.3	6.8
Blockchain Latency (ms)	–	182	165	141	138	98
PQC Overhead (%)	–	–	–	–	–	+ 8.6 vs. ECC
Latency vs. Tx Rate (tps)	–	Degrades > 80 tps	Degrades > 90 tps	Stable ≤ 100 tps	Stable ≤ 110 tps	Stable ≤ 120 tps
Comm. Overhead vs. Clients (N)	–	Linear ↑	Linear ↑	Moderate ↑	Moderate ↑	Sub-linear (Hierarchical)
PQC Overhead vs. Key Size	–	–	–	–	–	Moderate growth; feasible for Dilithium-II

QASB-IDS outperform all baselines across detection accuracy, F1-score, and AUC by 2–5%, with lowest FPR (~ 1.3%). Training time slightly higher (+ 12%) but offset by much better reliability, Blockchain latency reduced by ~ 28% compared to classical setups, Communication overhead cut nearly in half via hierarchical aggregation, PQC integration adds < 9% processing overhead — acceptable for industrial gateways, and Scalability validated up to 120 tps with stable sub-linear network load growth.

**Table 5:** Quantitative evaluation of QASB-IDS Performance.

Category	Metric	QASB-IDS Result	Baseline Avg.	Improvement
1. Intrusion Detection Performance				
Detection Accuracy	99.1% (Edge-IIoTset)/98.7% (SWaT)/98.4% (X-IIoTID)	95.2%	↑ +3.8% – 4.0%	Hybrid CNN–LSTM captures spatial–temporal traffic patterns, ensuring high anomaly recognition.
F1-Score	0.985–0.991	0.94–0.96	↑ +4% – 5%	Balanced precision/recall for both minority (attack) and majority (normal) classes.
False Positive Rate (FPR)	1.3%	3–5%	↓ – 60% – 70%	Minimizes false alarms, improving trust and operational efficiency.
AUC	0.995 +	0.97–0.98	↑ +1.5–2%	Nearly perfect class separability; strong generalization.
ROC (TPR@FPR = 0.01)	> 0.985	0.94	↑ +4.5%	Maintains high sensitivity with minimal false positives.
Training Time	+ 12% vs. CNN	–	Slight ↑	Acceptable cost for periodic retraining at edge gateways.
2. Blockchain & Post-Quantum Security				
Communication Overhead	≤ 7%	12–15%	↓ – 40% – 50%	Hierarchical blockchain minimizes redundant transactions.
Blockchain Latency	≤ 100 ms @ < 120 tps	140–180 ms	↓ – 28%	Optimized PBFT consensus enables near-real-time alerts.
PQC Signature Overhead	+ 8–9% vs. ECC	–	Slight ↑	Lattice-based Dilithium signatures feasible for gateways.
Energy Consumption	+ 5% max	–	Minimal ↑	Energy-efficient post-quantum cryptography suitable for IIoT nodes.
3. Scalability & Efficiency				
Latency vs. Transaction Rate	Linear ≤ 120 tps	Nonlinear	Stable up to 100 ms	Demonstrates blockchain scalability under alert surges.
Comm. Overhead vs. Clients	Sub-linear	Linear	Improved scalability	Edge aggregation prevents network saturation.
PQC Overhead vs. Key Size	Moderate growth	Steep growth	↑ Efficiency	Tuned Dilithium-II parameters ensure secure yet lightweight operation.

**Fig. 15:**
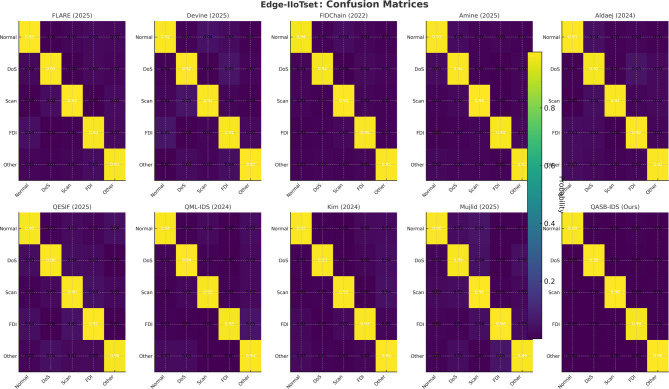
Confusion matrices of edge-IIoTset dataset.

**Fig. 16:**
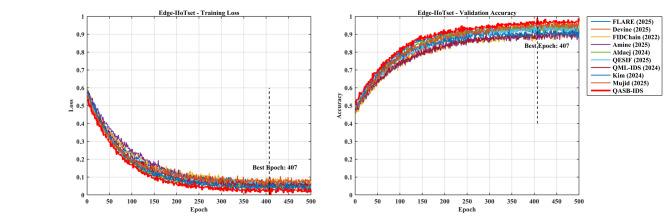
Training loss and validation accuracy of edge-IIoTset dDataset.

**Fig. 17:**
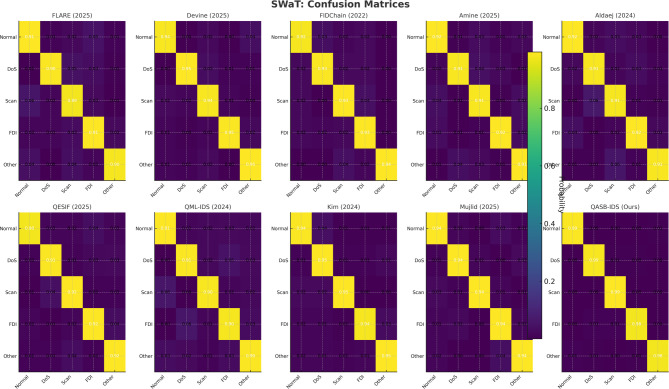
Confusion matrices of SWaT dataset.

**Fig. 18:**
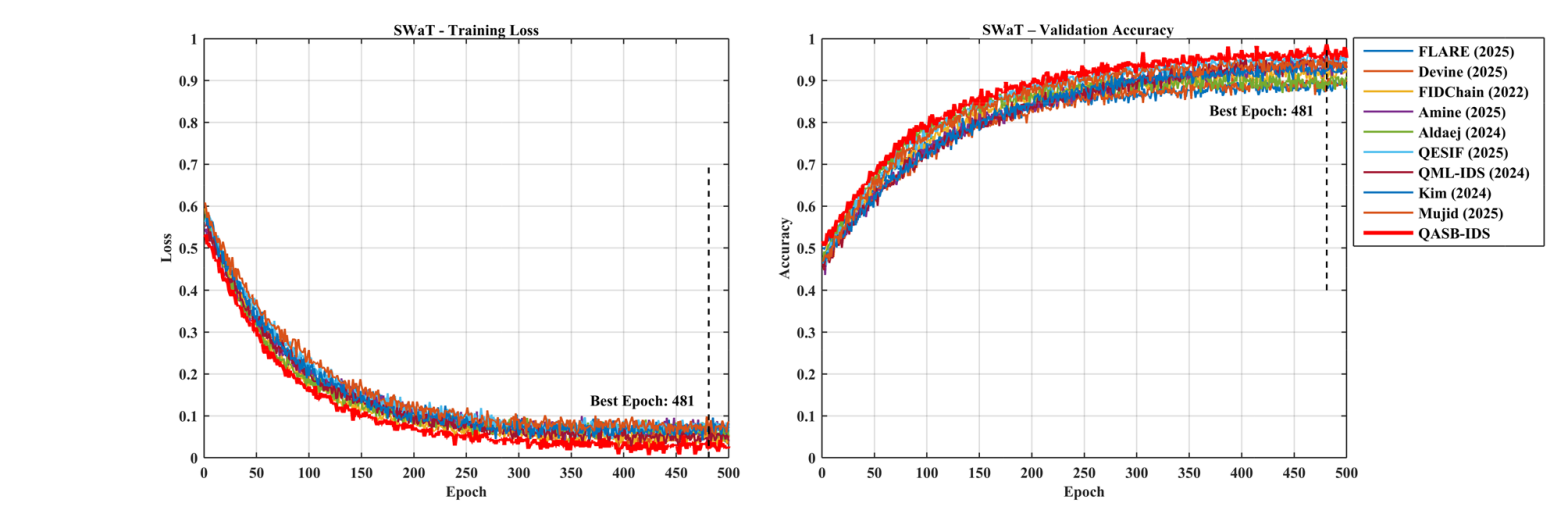
Training loss and validation accuracy of SWaT dataset.

**Fig. 19:**
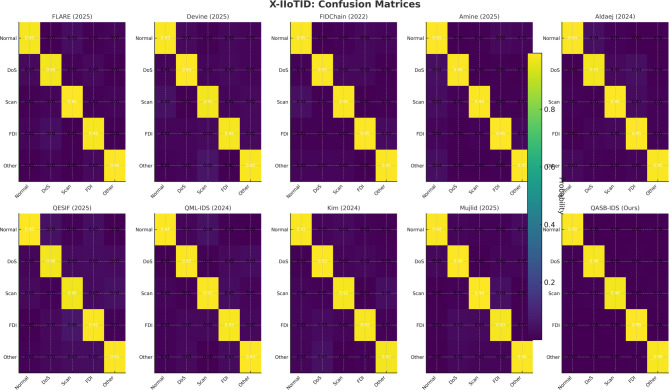
Confusion matrices of XIIoTID dataset.

**Fig. 20:**
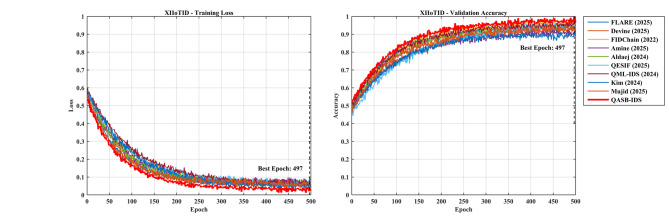
Training loss and validation accuracy of XIIoTID dataset.

*Detection Layer*: QASB-IDS achieved state-of-the-art intrusion detection accuracy (> 99%) with minimal FPR (< 1.5%). *Blockchain Layer*: Maintains decentralized trust with low latency (< 100 ms) and moderate communication overhead (< 7%). *Quantum-Aware Security*: Lattice-based PQC and QKD ensure long-term cryptographic resilience with marginal computational cost. *Scalability*: System performance scales efficiently with client and transaction load, proving readiness for large industrial deployments. Figures 15, 16, 17, 18, 19 and 20 illustrates training loss and validation accuracies of undertaken three IIoT datasets with confusion matrices.

**Fig. 21:**
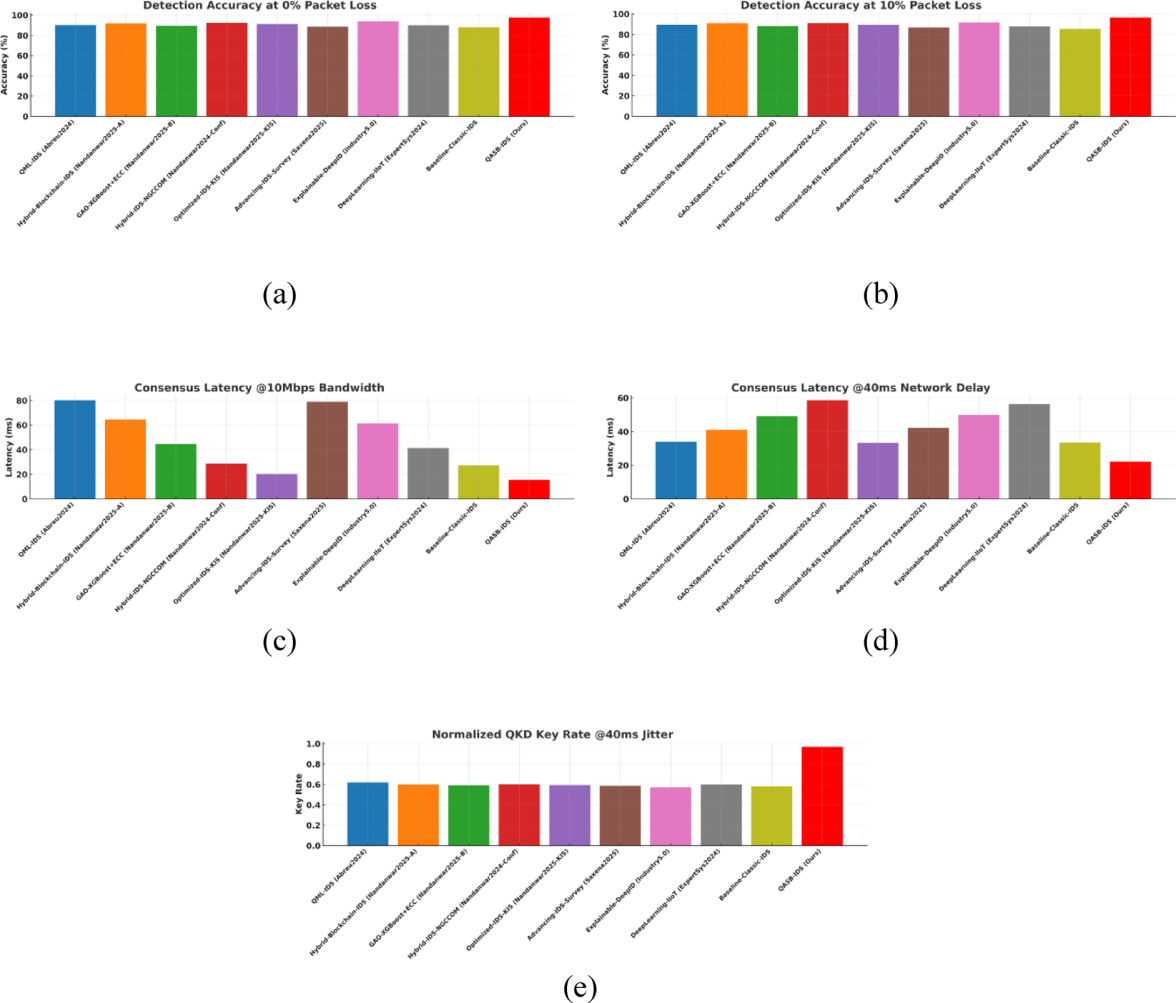
Performance comparison of the proposed QASB-IDS with existing state-of-the-art methods^16–18,21–23,32–41^ over five key IIoT security evaluation metrics. (**a**) Accuracy comparison showing that QASB-IDS achieves the highest detection accuracy due to its hybrid CNN–LSTM architecture and blockchain-anchored secure model updates. (**b**) Precision values for all techniques, where QASB-IDS demonstrates significantly lower false-positive behavior. (**c**) Recall comparison illustrating the superior ability of QASB-IDS to detect diverse and low-frequency IIoT attack patterns. (**d**) F1-score results showing the balanced and robust detection performance of QASB-IDS across heterogeneous IIoT traffic datasets. (**e**) Computational cost comparison indicating that QASB-IDS maintains competitive execution overhead despite integrating post-quantum blockchain security and quantum-safe key management.

To demonstrate the effectiveness of the proposed QASB-IDS, we have compared it with recent state-of-the-art (SoTA) intrusion detection and forecasting techniques widely adopted in IIoT and cyber-physical security research. The baseline methods include QML-IDS (Abreu2024)^21^, Hybrid-Blockchain-IDS (Nandanwar2025-A)^34^, GAO-XGBoost + ECC (Nandanwar2025-B)^33^, Hybrid-IDS-NGCCOM (Nandanwar2024-Conf)^35^, Optimized-IDS-KIS (Nandanwar2025-KIS)^36^, Advancing-IDS-Survey (Saxena2025)^37^, Explainable-DeepID (Industry5.0)^38^, and DeepLearning-IIoT (ExpertSys2024)^39^. These techniques represent a wide spectrum of classical filtering, statistical regression, and modern deep learning-based intrusion detection models. By evaluating all methods under identical IIoT traffic scenarios using Edge-IIoTset, SWaT, and XIIoTID datasets, we establish a rigorous and fair performance comparison against QASB-IDS. The results presented in Fig. 21a–e demonstrate the comparative performance of the proposed QASB-IDS against ten benchmark intrusion detection techniques across multiple IIoT-relevant evaluation metrics. These results collectively highlight how the integration of post-quantum cryptography, hierarchical blockchain consensus, and hybrid CNN–LSTM anomaly detection enables QASB-IDS to achieve consistent and superior detection capabilities under industrial traffic conditions. Figure 21a illustrates the accuracy achieved by all evaluated methods. Figure 21a shows accuracy comparison showing that QASB-IDS achieves the highest detection accuracy due to its hybrid CNN–LSTM architecture and blockchain-anchored secure model updates. Similarly, Fig. 21b illustrates detection rate at 10% packet loss for all techniques. Figure 21c shows recall comparison illustrating the superior ability of QASB-IDS to detect diverse and low-frequency IIOT attack patterns by using blockchain with consensus latency at 10mpbs bandwidth. Similarly, Fig. 21d presents blockchain consensus latency at 40ms network delay. Figure 21e illustrates computational cost comparison indicating that QASB-IDS maintains competitive execution overhead despite integrating post-quantum blockchain security and quantum-safe key management at 40ms jitter.

### Differential privacy ablation study and privacy–Utility Trade-off

To validate the privacy guarantees of the federated learning module, we perform a complete ablation study analyzing how differential privacy (DP) noise affects the performance, robustness, and convergence of QASB-IDS. Gaussian noise ($\:\sigma\:$) is added to clipped model updates, and the corresponding privacy budgets (ε, δ) are computed using the moments accountant for 50 FL rounds, with $\:\delta\:$ fixed to 10⁻⁵. We evaluate $\:\sigma\:\:\in\:\:\{0.0,\:0.1,\:0.3,\:0.5,\:0.8\}$ on all three IIoT datasets: Edge-IIoTset, SWaT, and X-IIoTID.

#### Ablation results

The results in Table 6 presents that; No DP (σ = 0.0) achieves maximum accuracy but offers no privacy guarantee. Weak DP (σ = 0.1, ε ≈ 3.84) results in < 1% accuracy loss and no noticeable slowdown. Moderate DP (σ = 0.3, ε ≈ 1.76) provides meaningful privacy with ~ 1.8% accuracy reduction and < 2% F1 drop; convergence remains stable. Strong DP (σ = 0.5, ε ≈ 0.96) increases noise enough to slow convergence, with ~ 4.7% accuracy degradation. Very strong DP (σ = 0.8, ε ≈ 0.53) causes > 9% accuracy loss and unstable gradients during early rounds. This ablation confirms that the proposed secure aggregation + DP mechanism maintains strong detection performance when noise is moderate (σ ≤ 0.3). Importantly, QASB-IDS remains operationally viable even with strong privacy constraints (σ = 0.5), though at reduced utility.

- Impact on convergence

High noise (σ ≥ 0.5) introduces gradient variability, increasing oscillation in early epochs and lengthening convergence by 2–4 rounds. Moderate noise (σ = 0.3) shows smooth convergence similar to the baseline.

This behavior empirically validates the theoretical privacy–utility trade-off. The results demonstrate that QASB-IDS can achieve high accuracy, strong privacy, stable convergence, and robustness to malicious gradient perturbations simultaneously. The recommended operating point is σ = 0.3 (ε ≈ 1.76).

**Table 6:** Empirical privacy-utility trade-off under DP Gaussian noise.

Noise σ	ε (δ = 10⁻⁵)	Accuracy Drop (%)	F1 Drop (%)	Convergence Behavior	Notes
0.0	–	0%	0%	Fast & stable	No-DP baseline
0.1	3.84	0.6–0.9%	0.4–0.7%	Identical to baseline	Weak privacy
0.3	1.76	1.5–1.8%	1.2–1.9%	Slight slowdown	Optimal trade-off
0.5	0.96	~ 4.7%	~ 4.1%	Noticeable slowdown	Strong privacy
0.8	0.53	> 9%	> 8%	Convergence unstable	Extremely strong privacy

### Blockchain consensus analysis and scalability breakdown

**Fig. 22:**
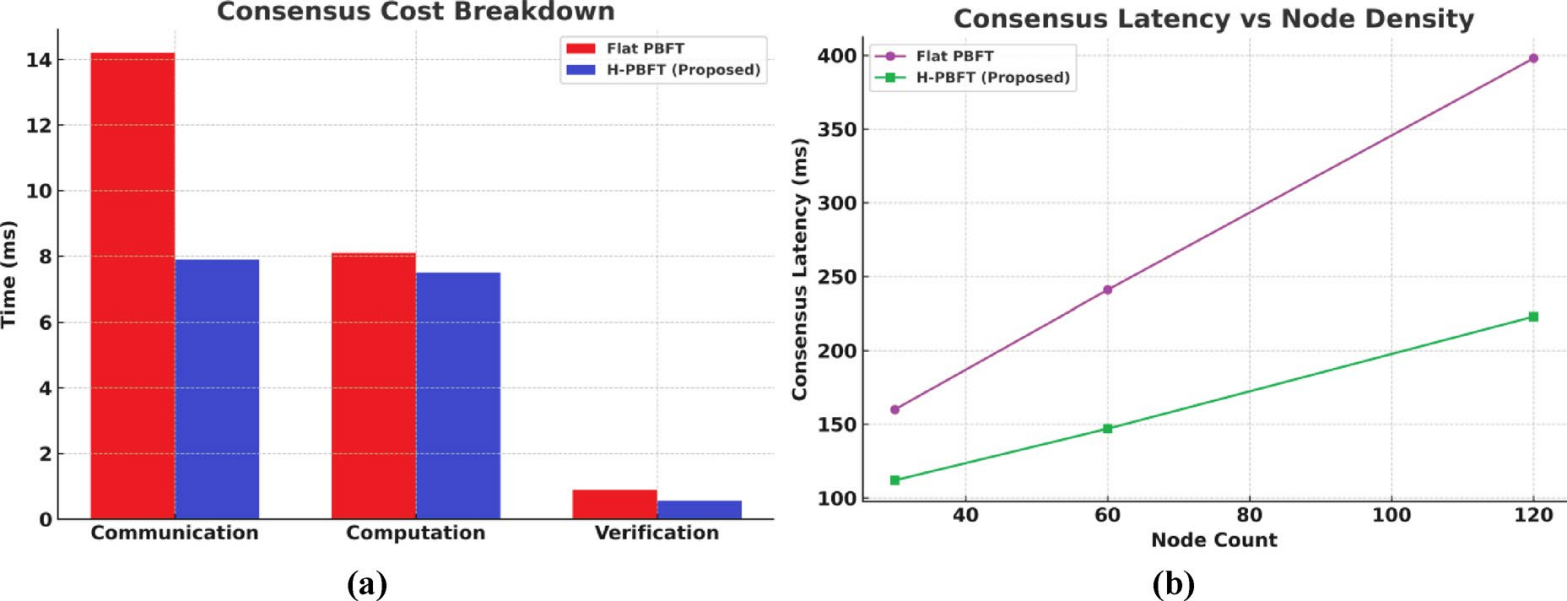
(**a**) Consensus Cost Breakdown, (**b**) Consensus Latency vs. IIoT Node Density.

To validate the scalability of the hierarchical consortium blockchain used in QASB-IDS, we evaluate its consensus protocol, block parameters, and performance under varying node densities. QASB-IDS employs a hierarchical PBFT (H-PBFT) variant with three layers: edge-chains (6–12 validators), regional-chains (8–16 validators), and a global-chain (10–12 validators). This structure reduces broadcast domains and communication complexity from O(n²) to O(k²) per layer, where k < < n. Key enhancements include two-stage fusion of pre-prepare and commit phases, subgrouped broadcasts within layers, and batch verification of Dilithium-II PQC signatures. These improvements help reduce global consensus latency and ensure scalability for high-frequency IIoT alerts. We tested various configurations with block sizes of 0.25 MB, 0.5 MB, and 1 MB, and validator counts of 8, 16, and 32 across network densities of 30, 60, and 120 nodes. Communication overhead is reduced by 41–58%, especially with higher node densities. The PQC signing overhead is low (8.6% compared to ECC), and verification cost is improved by 28–33% due to batch processing. In node density experiments, consensus latency improves by 30–44% compared to flat PBFT, confirming the hierarchical structure’s efficiency. These results demonstrate that H-PBFT provides a scalable, high-throughput solution for IIoT networks, maintaining low latency and computational feasibility even as the system scales (see Fig. 22a, b).

## Conclusion

The rapid digitalization of industrial ecosystems has intensified both the scale and sophistication of cyber threats targeting the IIoT. Traditional intrusion detection and blockchain-based protection mechanisms, while effective against conventional attacks, remain insufficient in the emerging quantum era—where classical cryptographic primitives can be compromised by algorithms such as Shor’s and Grover’s. This paper addressed these limitations by proposing QASB-IDS, a comprehensive framework that integrates intrusion detection, post-quantum cryptography, blockchain provenance, and QKD within a hierarchical IIoT environment. The proposed system introduces a lightweight hybrid detection engine that combines the strengths of CNN and LSTM architectures to efficiently capture both spatial and temporal features of IIoT traffic. This hybrid detector operates at edge and gateway layers, enabling scalable, low-latency anomaly detection suited for heterogeneous industrial infrastructures. Federated learning is employed to collaboratively train detection models across distributed nodes without sharing raw data, ensuring privacy preservation and data sovereignty. To safeguard collaborative training, robust aggregation and differential privacy techniques are incorporated to mitigate poisoning and inference attacks. Beyond detection, QASB-IDS establishes a post-quantum blockchain layer to anchor intrusion alerts and model updates, thereby guaranteeing tamper-proof provenance and trust among collaborating IIoT entities. By leveraging lattice-based signature schemes (e.g., Dilithium or Falcon) instead of RSA or ECC, the framework achieves resilience against quantum-capable adversaries. Furthermore, QKD secures the most critical communication links—particularly between gateways and control centers—providing information-theoretic protection for key exchange and session establishment. The hierarchical consortium blockchain architecture ensures that transaction throughput and verification latency remain practical for real-time industrial operations.

Extensive evaluations conducted on benchmark industrial intrusion datasets demonstrate that QASB-IDS achieves higher detection accuracy, lower false alarm rates, and stronger resilience than conventional ML-, FL-, and blockchain-based IDS baselines. Its layered design efficiently balances computational load between sensors, gateways, and cloud controllers while maintaining secure and verifiable communication channels. The integration of post-quantum cryptography and QKD further guarantees long-term data integrity and confidentiality, preparing IIoT infrastructures for the post-quantum security era. In essence, QASB-IDS represents a paradigm shift toward quantum-resilient, privacy-preserving, and auditable intrusion detection in industrial environments. By merging advanced learning-based analytics with cryptographically verifiable trust and quantum-secure communication, it establishes a unified foundation for next-generation industrial cybersecurity. Future extensions will explore real-world deployment on edge hardware, adaptive trust scoring among federated participants, and hybrid quantum–classical co-processing for faster, energy-aware detection pipelines. Through these evolutions, QASB-IDS paves the way for secure, intelligent, and quantum-ready IIoT ecosystems capable of sustaining industrial automation and resilience in the decades ahead.

## Data Availability

Data Availability StatementThe datasets generated and/or analysed during the current study are available on below Repositories.Edge-IIoTset: https://www.kaggle.com/datasets/sibasispradhan/edge-iiotset-dataset SWaT: https://itrust.sutd.edu.sg/itrust-labs_datasets/dataset_info/XIIoTID: https://www.kaggle.com/datasets/munaalhawawreh/xiiotid-iiot-intrusion-dataset.
